# Supplementary material for: Therapeutic validation of MMR-associated genetic modifiers in a human ex vivo model of Huntington disease
Source: Am J Hum Genet. 2024 May 14;111(6):1165–83. doi: 10.1016/j.ajhg.2024.04.015 (PMC11179424; doi:10.1016/j.ajhg.2024.04.015)
Supplement: Document S1. Figures S1–S7 and Tables S1–S5 [file mmc1.pdf]

The American Journal of Human Genetics, Volume *111*

Supplemental information

Therapeutic validation of MMR-associated genetic modifiers in a human *ex vivo* model of Huntington disease

Ross Ferguson, Robert Goold, Lucy Coupland, Michael Flower, and Sarah J. Tabrizi

SUPPLEMENTAL MATERIALS

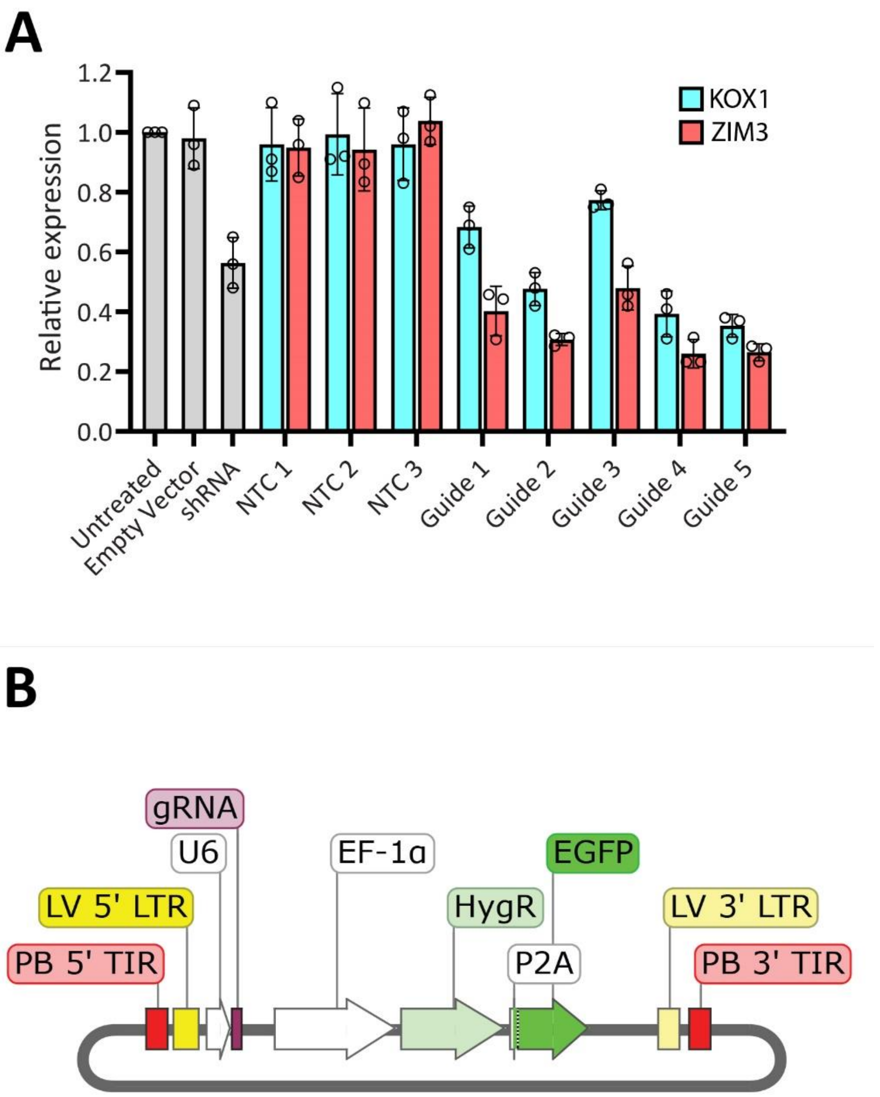

Figure S1 – A more effective repressor construct and guide delivery vector. Comparison between KOX1 and ZIM3 KRAB domains indicated better or same knock-down using PMS2 targeting guides (A). Guide sequences were cloned into either pLentiguide GFP-HYG or a modified version with additional PiggyBAC terminal inverted repeats (B).

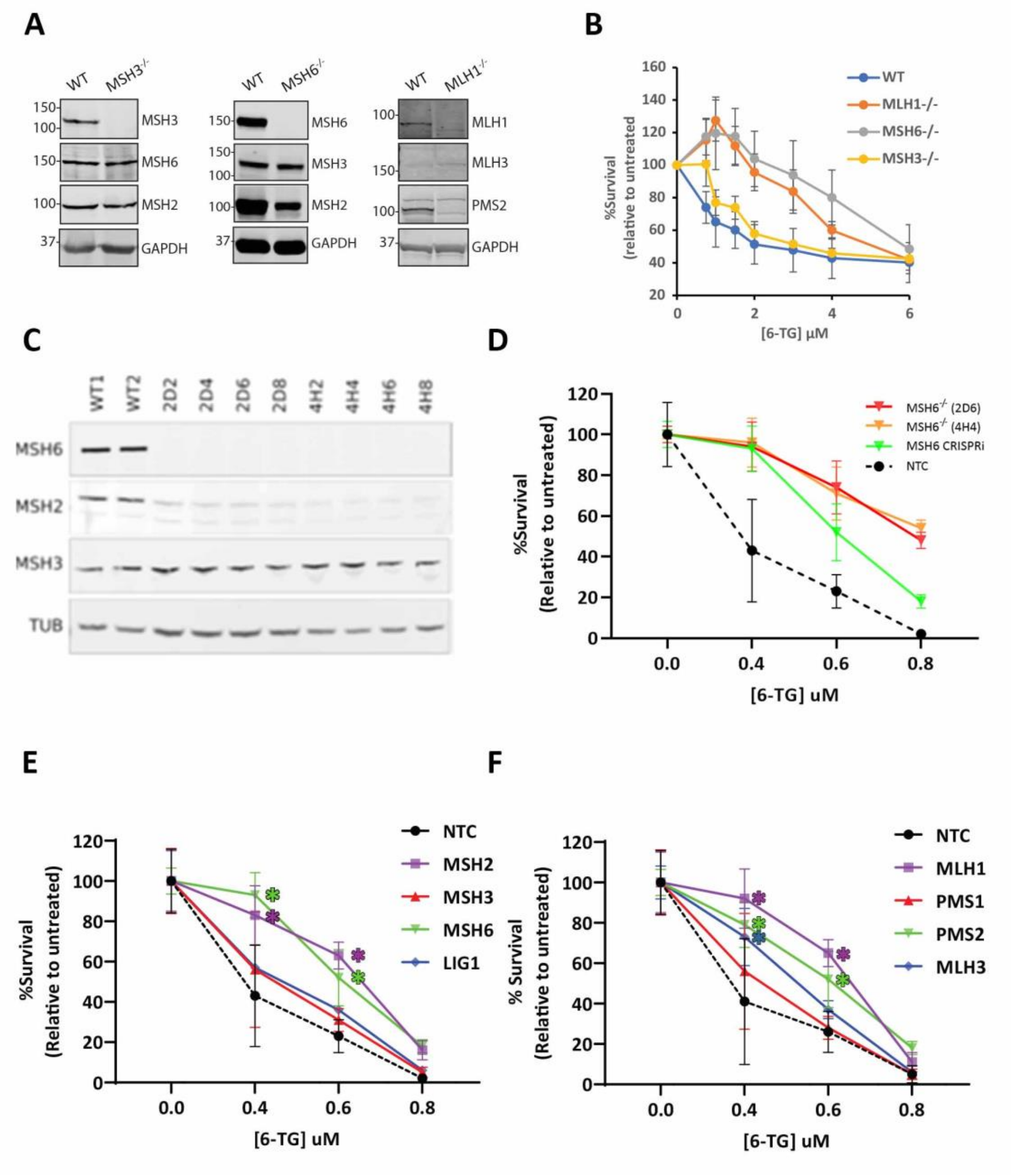

Figure S2 – Knockout of MMR factors in U2OS cells results in MMR deficiency.

Western blot for MMR associated proteins in U2OS MSH3, MSH6 & MLH1 knockout clones (-/-), compared to wild type U2OS (WT) (A). MMR deficiency seen after 6-thioguanine treatment of MSH3, MSH6 and MLH1 null and wildtype U2OS cells (B). Western blot of MSH6 knockout 125Q iPSCs generated using the same strategy with deletions in exon 2 or 4 (C). MSH6 knockout iPSC (clones 2D6 & 4H4) show further reduced sensitivity to 6-TG treatment than CRISPRi lowered MSH6 iPSCs (D). iPSCs show high vulnerability to 6-TG with a small window for seeing functional differences between genotype in MutS (E), or MutL (F) CRISPRi iPSCs. *P<0.05.

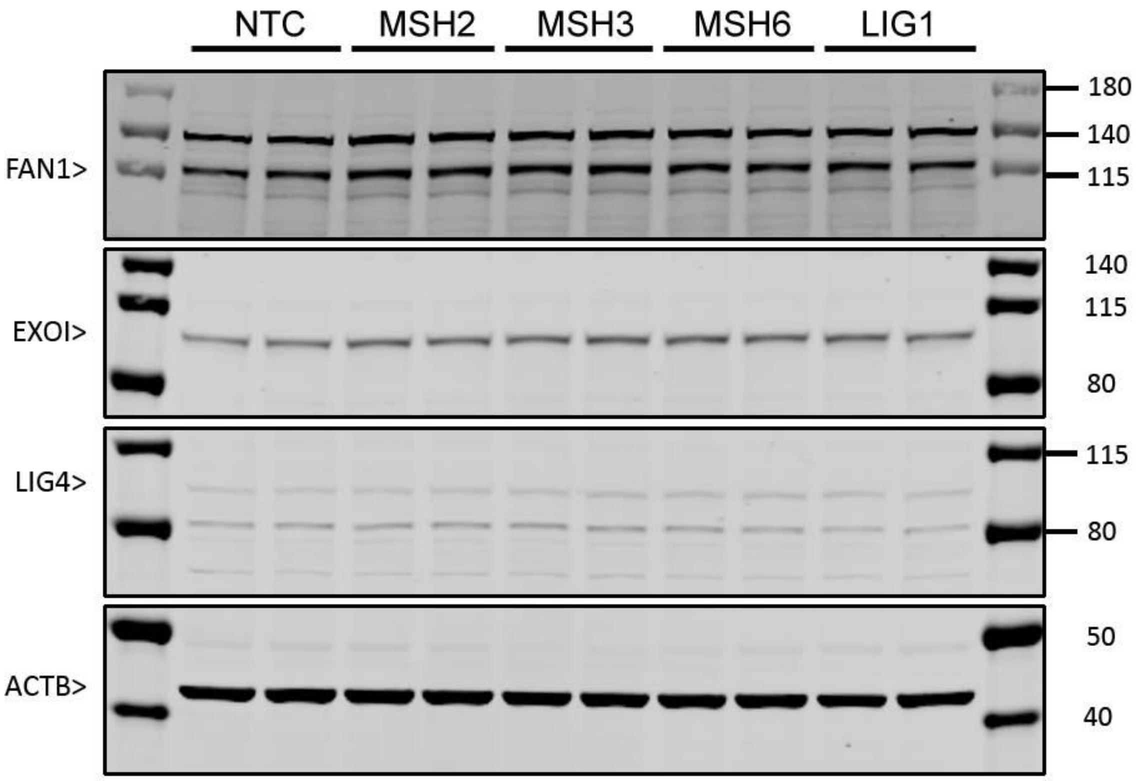

Figure S3 – No changes seen in LIG4, EXO1 or FAN1 in CRISPRi MutS pools. Western blots for indicated proteins (left) in two different CRISPRi pools targeting the indicated MutS factors and LIG1.

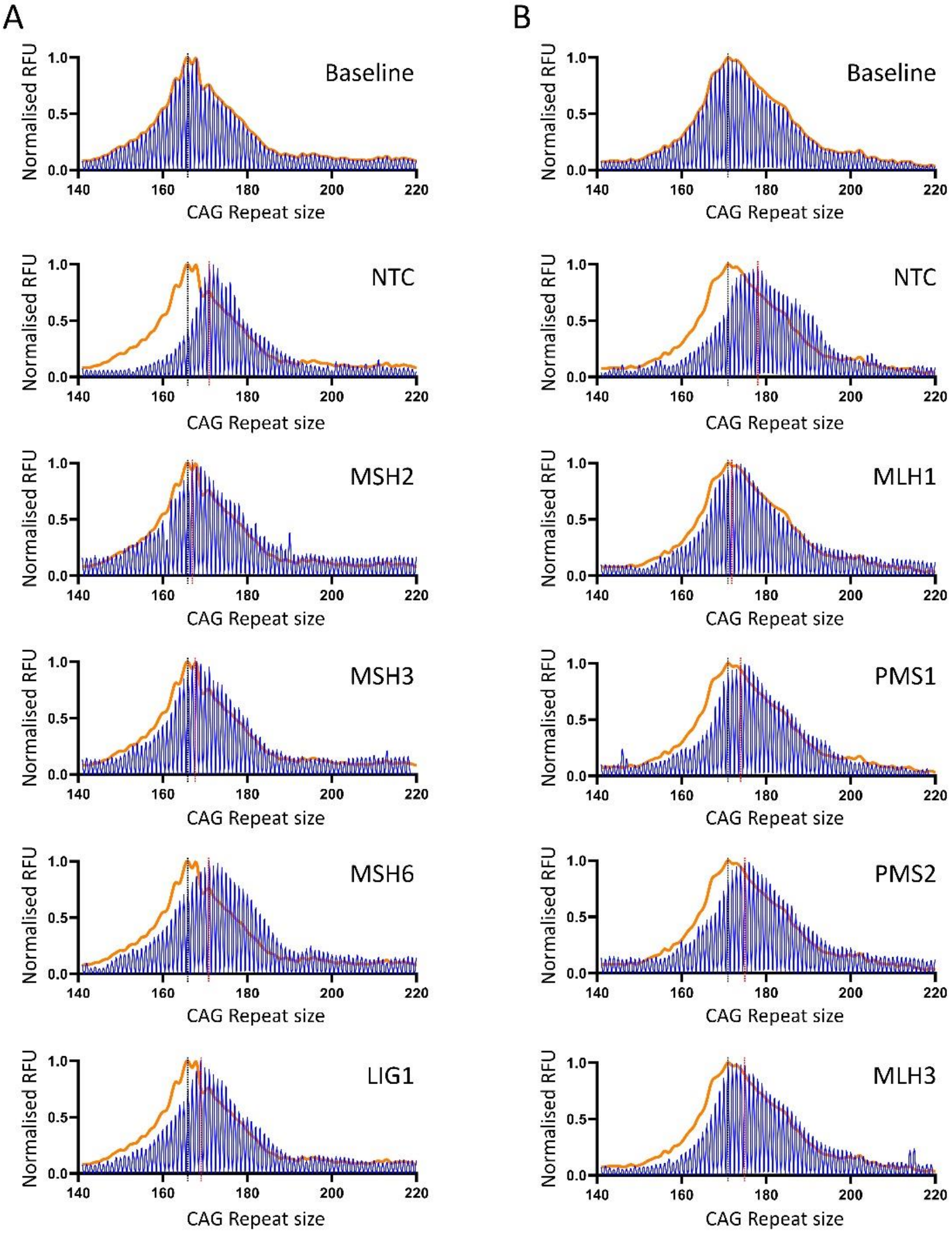

Figure S4 – Representative fragment analysis traces for CRISPRi iPSCs.

Representative traces with peak height normalised to the modal peak for baseline samples (D0) or endpoint (D80) for each of the CRISPRi pools where MutS & LIG1 (A), or MutL (B) are lowered. Dashed black vertical lines show modal baseline CAG size. Dashed red vertical lines show modal CAG size at endpoint. Orange trace shows the baseline from each pool for reference in each panel

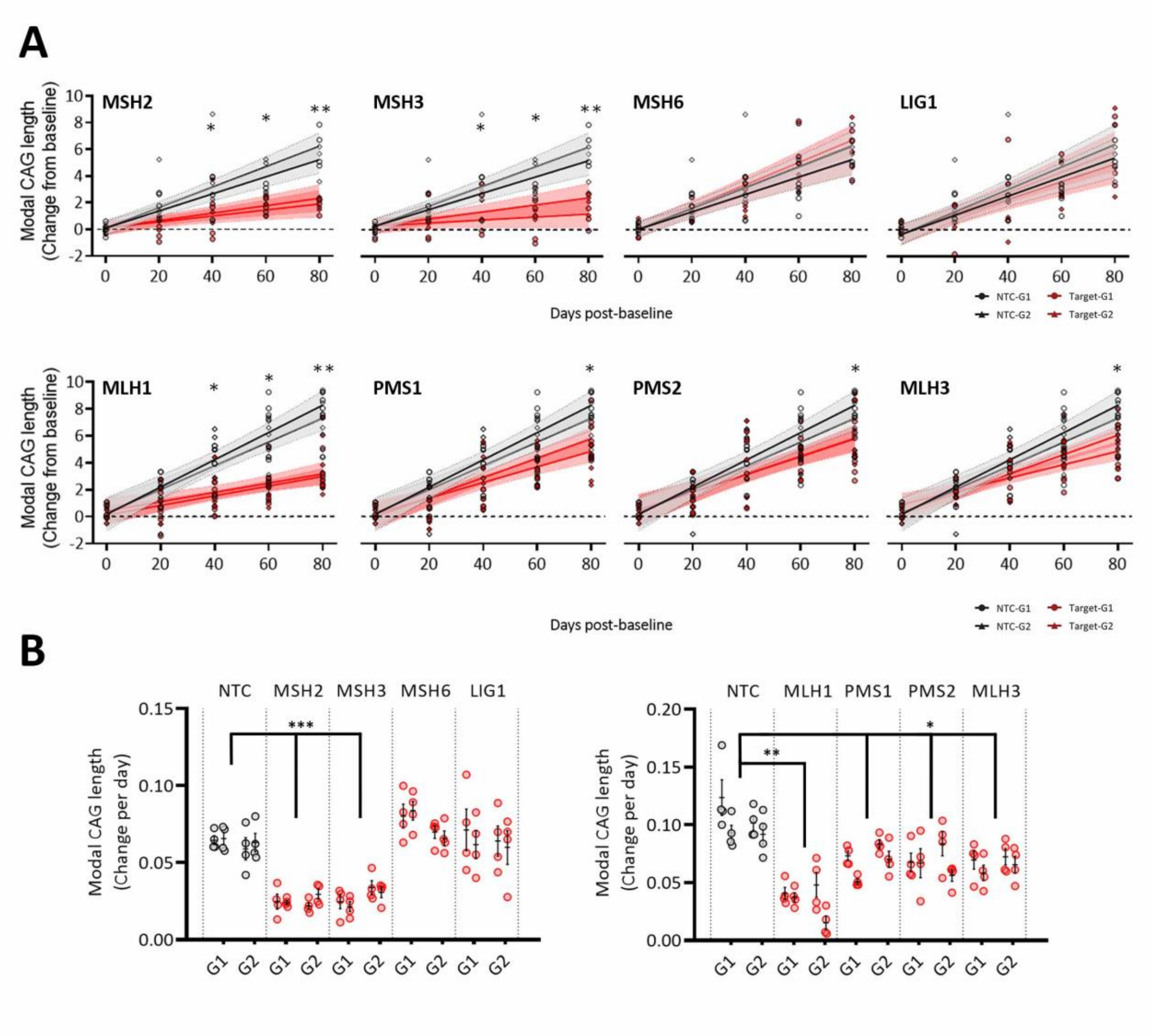

Figure S5 - Reduced expression of MutS and MutL components slows increase in modal repeat length in dividing iPSCs. Change in modal CAG repeat length over an eighty-day period for MutS & LIG1 (A), and MutL (B) lowered cultures relative to baseline on D0. Four cultures per guide passaged in parallel each from two independent CRISPRi pools, dashed lines 95% confidence intervals. Rate of change in modal repeat length per day for MutS & LIG1 (C), and MutL (D). Open circles represent parallel cultures with mean bar ±SEM. * P<0.05 * P<0.005.

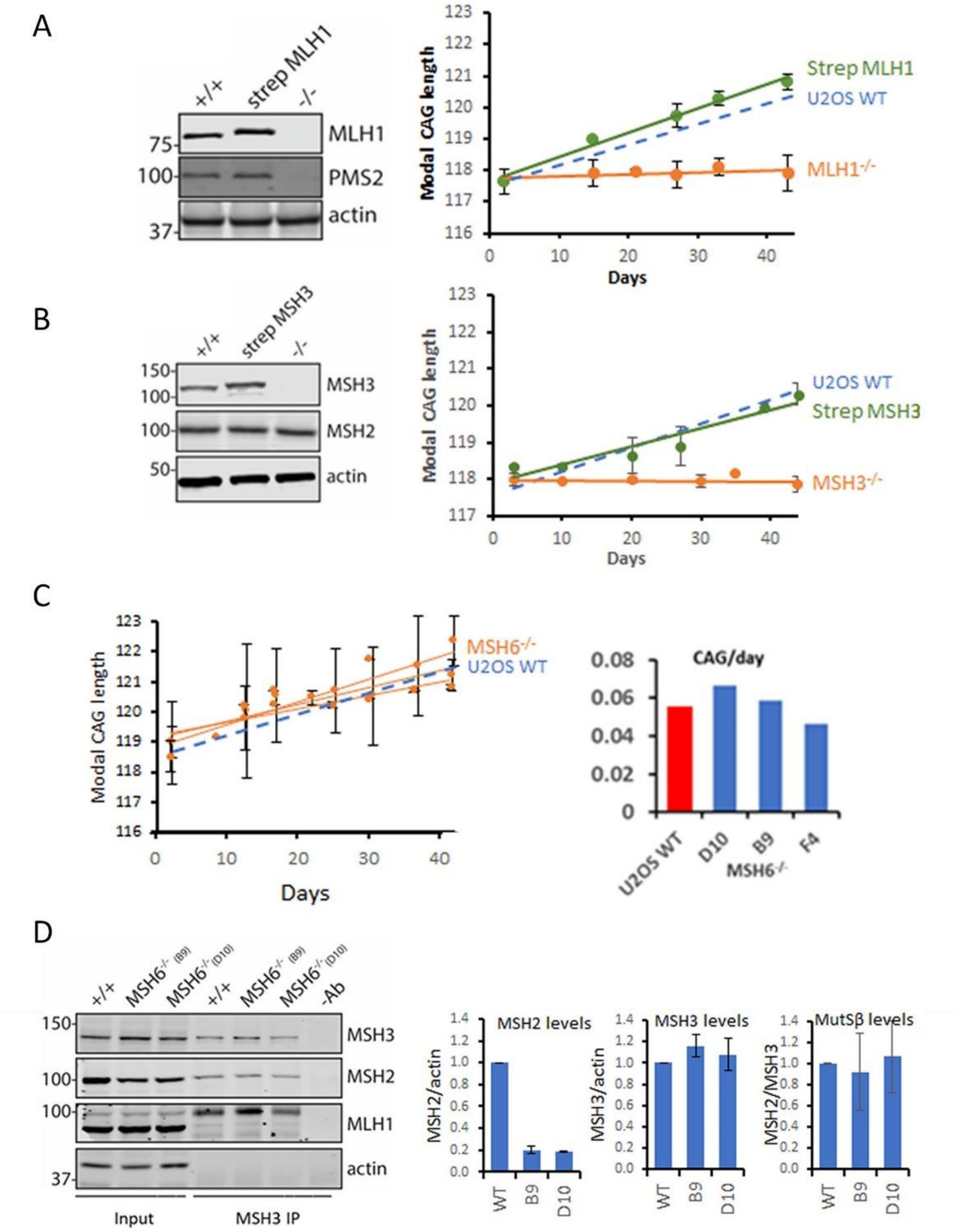

Figure S6 – Repeat expansion in MSH3, MLH1 and MSH6 null U2OS. A 118Q repeat construct introduced by transduction shows an increase in modal length over 40 days. Expansion is halted in $MLH1^{-/-}$ U2OS cells but can be rescued to WT U2OS cells rates by adding back a strep-tagged MLH1 to near physiological levels by dox-inducible expression (A). Expansion is halted and rescued in $MSH3^{-/-}$ U2OS cells using the same system (B). No change repeat expansion is observed in $MSH6^{-/-}$ U2OS cells (C). Immunoprecipitation shows that while MSH2 levels are lowered in $MSH6^{-/-}$ cells there is no change in MSH3/MSH2 interactions (D)

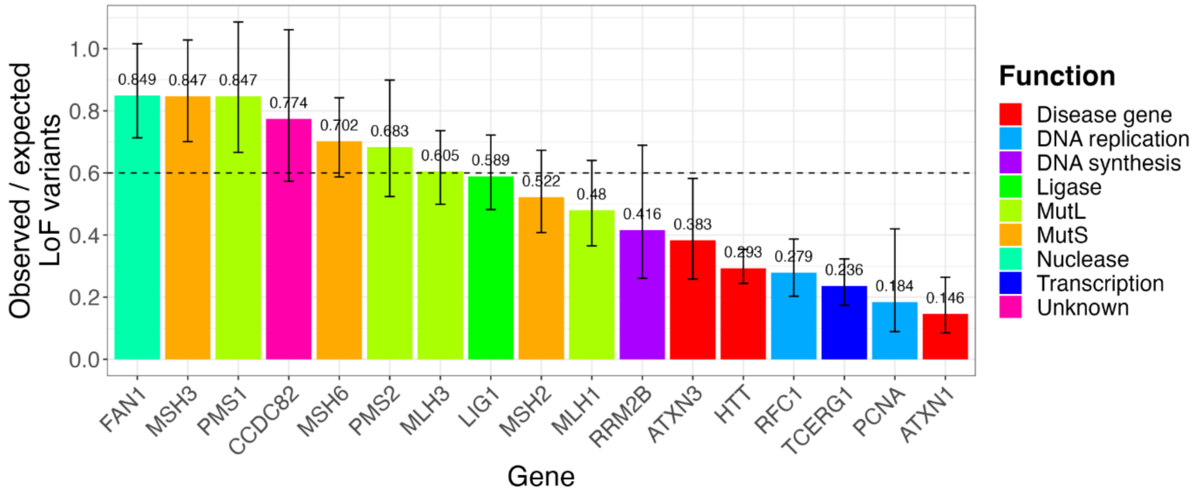

Figure S7 – Genetic constraint in selected HD modifier and DNA repair genes. Bar chart of loss-of-function metrics for selected genes, based on data from gnomAD (Gudmundsson et al., 2022). The y-axis represents the ratio of observed / expected ratio of loss-of-function variants (pLoF) for each gene, using GENCODE v39 annotations and GRCh38 reference. Error bars represent the 90% confidence interval. Lower o/e values indicate genes are under stronger selection. An o/e ratio of 0.2, for example, means there were 20% of the expected number of variants observed in that gene. Bars falling below the horizontal dashed line at an o/e ratio of 0.6 are generally considered to be under a high degree of genetic constraint, with significant selection against loss-of-function variation.

Table S1 – CRISPRi guide sequences

Target	Guide	Sequence (5'-3')
**NTC**	1	TCTCTCGGAGTGGAGCAACA
**NTC**	2	GTCAGGTAATAGTCGGACTC
**NTC**	3	CGCAATCCCTTAGGATAGCC
**NTC**	4	ATCTACAATCCAGCCCTCTA
**MSH2**	1	GAGGTGAGGAGGTTTCGACA
**MSH2**	2	GTGAGGAGGTTTCGACATGG
**MSH2**	3	CGACATGGCGGTGCAGCCGA
**MSH2**	4	GACGCTGCAGTTGGAGAGCG
**MSH3**	1	AGGATGGCAGCCCGGCGGCA
**MSH3**	2	GGGCAAGGATGGCAGCCCGG
**MSH3**	3	GAGACATGGCAGGGCAAGGA
**MSH3**	4	GCTTCCGGCGAGACATGGCA
**MSH6**	1	AAAGCACCGCATCTACCGCG
**MSH6**	2	CAGGAGCCGCGCGGTAGATG
**MSH6**	3	GGCCCAACCGTTCTGTCGGA
**MSH6**	4	GCTCCGTCCGACAGAACGGT
**LIG1**	1	GCAGTCCCAAGTTCGCGCCA
**LIG1**	2	GAATGCCGTGGCGCGAACTT
**LIG1**	3	GCGCGAACTTGGGACTGCAG
**LIG1**	4	GCAACACACTCAGATCCGCC
**MLH1**	1	TCGGCGGCTGGACGAGACAG
**MLH1**	2	CCAAAATGTCGTTCGTGGCA
**MLH1**	3	CAAAATGTCGTTCGTGGCAG
**MLH1**	4	TCGTGGCAGGGGTTATTCGG
**MLH1**	5	TGGCGCCAAAATGTCGTTCG
**PMS1**	1	ACTACCTTCCTGCTAGCGCG
**PMS1**	2	GCGCTAGCAGGAAGGTAGTG
**PMS1**	3	ATTGCCTGCCTCGCGCTAGC
**PMS1**	4	GGCTGCTTGCGGCTAGTGGA
**PMS1**	5	CGCTGGCTGCTTGCGGCTAG
**PMS2**	1	CGGATCGGGTGTTGCATCCA
**PMS2**	2	AGCTGAGAGCTCGAGGTGAG
**PMS2**	3	CGAGCTCTCAGCTCGCTCCA
**PMS2**	4	ACGAGATCGCTGCAACACTG
**PMS2**	5	AACACTGAGGTCGCCACTCC
**MLH3**	1	AAGATCCAAGGTGCGCGCGT
**MLH3**	2	GTGTCGGAGAATTTGTTAAG
**MLH3**	3	TGTCGGAGAATTTGTTAAGC
**MLH3**	4	TTCTCCGACACCAACCGCCT
**MLH3**	5	TTCTCCGACACCAACCGCCT

Table S2 – Primer sequences

Primer	Sequence (5'-3')	Amplicon
**pLG_cPCR_F1**	TTTCAGACCCACCTCCCAAC	2447bp +Stuffer
**pLG_cPCR_R1**	AGTGGATCTCTGCTGTCCCT	558bp - Stuffer
**pLG_cPCR_F2**	ATCGTTTCAGACCCACCTCC	2242bp +Stuffer
**pLG_cPCR_R2**	ATCGTTTCAGACCCACCTCC	353bp - Stuffer
**PB 5P TIR - pLG**	cttcccaacagttgcCATGCGTCAATTTTACGCATGATTATCTTTAACGTACGTCACAATATGATTATCTTTCTAGGGttaaagcctgaatggcgaatgg	1751bp
**PB 3P TIR - pLG**	ttcgccagttaatagtttgcCATGCGTCAATTTTACGCAGACTATCTTTCTAGGGTTAAgtctgacgctcagtggaacg	
**ZIM3_5P_HindIII**	cacaagcttaattctggctaactgtcggg	398bp
**ZIM3_3P_Stop_MluI**	aattcacgcgtctaaaccactttgtacaagaaagttgggtagag	
**ZNF80_F1**	TGCAGCTCATCCTCACTTGG	436bp
**ZNF80_R1**	GAGGCAAGGCCTTTGTACCT	
**GPR15_F1**	CTTGCATGAGTGTTGACCGC	303bp
**GPR15_R1**	AATGGGCACACAGCTTCCTT	
**C13_5PJ_F1**	TATCGGTATCCTCGACTTGGC	1843bp
**C13_5PJ_R1**	CTCCTCCACGTCACCGCA	
**C13_3PJ_F1**	GCAACCTGTTCAGTGCGTC	1975bp
**C13_3PJ_R1**	AGTATGCTTATGCCAAGCCA	
**dCas9_F1**	CATCGAGCAGATCAGCGAGT	275bp
**dCas9_R1**	CGATCCGTGTCTCGTACAGG	
**T7_MSH6_Ex2_F1**	GCCTTTTTCCTGCCATCAGC	647bp
**T7_MSH6_Ex2_R1**	TTTCACAACTGCCACCCCTT	
**T7_MSH6_Ex4_F1**	TTGGCATATGAAGTTGCAGCATA	642bp
**T7_MSH6_Ex4_R1**	GCTGTTCAGGCCTTCACTCT	

Table S3 – Knockout guides

MSH6 (NG_007111.1)	Sequence (5'-3')	Cut position
**Hs.Cas9.MSH6.1.AF**	AGCCUAAGACACAAGGAUCUGUUUUAGAGCUAUGCU	20548
**(Targeting)**	AGCCTAAGACACAAGGATCTAGG	
**Hs.Cas9.MSH6.1.AH**	AUUUAAGCCAGACACUAAGGGUUUUAGAGCUAUGCU	20645
**(Targeting)**	ATTTAAGCCAGACACTAAGGAGG	

Table S4 – TaqMan Assays

Target	Assay ID
**ATP5B**	Hs00969569_m1
**EIF4A2**	Hs00756996_g1
**UBC**	Hs00824723_m1
**MLH1**	Hs00179866_m1
**PMS1**	Hs00922262_m1
**PMS2**	Hs00241053_m1
**MLH3**	Hs00998142_m1
**MSH2**	Hs00953527_m1
**MSH3**	Hs00989003_m1
**MSH6**	Hs00943000_m1
**LIG1**	Hs01553527_m1

Table S5 – Antibodies

Primary	Supplier	Cat#	Dilution
**MLH1**	BD	#554073	1:500 (ICC), 1:1000 (WB)
**PMS1**	Invitrogen	PA5-86724	1:500 (ICC), 1:1000 (WB)
**PMS2**	Invitrogen	PA5-87127	1:500 (ICC), 1:1000 (WB)
**MLH3**	Santa Cruz	sc-25313	1:500 (ICC), 1:1000 (WB)
**MLH3**	ProteinTech	25298-1-AP	1:500 (ICC), 1:1000 (WB)
**MSH2**	Cell Signalling Technologies	#2017	1:500 (ICC), 1:1000 (WB)
**MSH3**	ProteinTech	22393-1-AP	1:500 (ICC), 1:1000 (WB)
**MSH6**	BD	610918	1:500 (ICC), 1:1000 (WB)
**LIG1**	ProteinTech	18051-1-AP	1:500 (ICC), 1:1000 (WB)
**FAN1**	University of Dundee / CHDI	FS2	1:2000 (WB)
**EXO1**	ProteinTech	16253-1-AP	1:2000 (WB)
**LIG4**	ProteinTech	12695-1-AP	1:2000 (WB)
**ACTB**	Abcam	ab8226	1:5000 (WB)
**TUB**	Abcam	ab6046	1:5000 (WB)
**GAPDH**	Abcam	ab9485	1:5000 (WB)
**CTIP2**	Abcam	ab18465	1:500 (ICC), 1:1000 (WB)
**DARPP-32**	Cell Signalling Technologies	#2306	1:500 (ICC)
**MAP2**	Novus	NB300-213	1:10,000 (ICC)
**NFL**	Cell Signalling Technologies	#2837	1:2000 (WB)
**TUBB3**	Abcam	ab41489	1:2000 (ICC)
**NEUN**	Abcam	ab104224	1:500 (ICC), 1:1000 (WB)
**PCNA**	Cell Signalling Technologies	#2586	1:500 (ICC), 1:2000 (WB)
**OCT4**	Santa Cruz	sc-5279	1:1000 (ICC), 1:1000 (WB)
**NANOG**	abcam	ab21624	1:1000 (ICC)
**SSEA4**	Invitrogen	MA1-021	1:1000 (ICC)
**LIN28**	Cell Signalling Technologies	#3978	1:1000 (ICC)

Secondary	Supplier	Cat#	Dilution
**IRDye 800CW Goat anti-Mouse (H+L)**	LiCor	926-32210	1:15000
**IRDye 680RD Goat anti-Rabbit (H+L)**	LiCor	926-68071	1:15000
**AlexaFluor 488 Gt α Ms**	Invitrogen	A-11001	1:2000
**AlexaFluor 594 Gt α Rbt**	Invitrogen	A-11008	1:2000
**AlexaFluor 488 Gt α Rbt**	Invitrogen	A-11012	1:2000
**AlexaFluor 568 Gt α Rbt**	Invitrogen	A-11011	1:2000
**AlexaFluor 488 Gt α Rt**	Invitrogen	A-11006	1:2000
**AlexaFluor 647 Dk α Ms**	Invitrogen	A-31571	1:2000
**Hoechst 33342**	Invitrogen	H3570	1:2000
