# Supplementary material for: Therapeutic validation of MMR-associated genetic modifiers in a human ex vivo model of Huntington disease
Source: Am J Hum Genet. 2024 May 14;111(6):1165–83. doi: 10.1016/j.ajhg.2024.04.015 (PMC11179424; doi:10.1016/j.ajhg.2024.04.015)
Supplement: Document S2. Article plus supplemental information [file mmc2.pdf]

Therapeutic validation of MMR-associated genetic modifiers in a human *ex vivo* model of Huntington disease

Graphical abstract

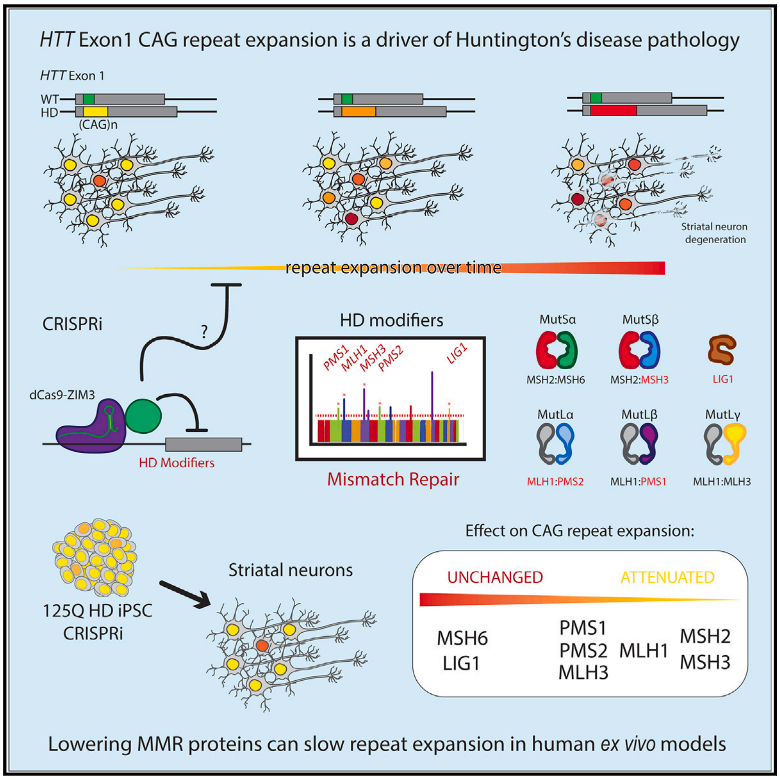

Authors

Ross Ferguson, Robert Goold, Lucy Coupland, Michael Flower, Sarah J. Tabrizi

Correspondence

s.tabrizi@ucl.ac.uk

The expanded trinucleotide repeat underlying Huntington disease (HD) continues to lengthen across the carrier's lifetime and correlates with disease onset. A core of DNA mismatch repair factors has been identified as onset modifiers, and we show that lowering expression of these factors slows repeat expansion in striatal neurons derived from HD iPSCs.

Ferguson et al., 2024, The American Journal of Human Genetics *111*, 1165–1183
June 6, 2024 © 2024 The Author(s).
https://doi.org/10.1016/j.ajhg.2024.04.015

Therapeutic validation of MMR-associated genetic modifiers in a human *ex vivo* model of Huntington disease

Ross Ferguson,[1,2] Robert Goold,[1,2] Lucy Coupland,[1,2] Michael Flower,[1,2] and Sarah J. Tabrizi[1,2,*]

Summary

The pathological huntingtin (*HTT*) trinucleotide repeat underlying Huntington disease (HD) continues to expand throughout life. Repeat length correlates both with earlier age at onset (AaO) and faster progression, making slowing its expansion an attractive therapeutic approach. Genome-wide association studies have identified candidate variants associated with altered AaO and progression, with many found in DNA mismatch repair (MMR)-associated genes.

We examine whether lowering expression of these genes affects the rate of repeat expansion in human *ex vivo* models using HD iPSCs and HD iPSC-derived striatal medium spiny neuron-enriched cultures. We have generated a stable CRISPR interference HD iPSC line in which we can specifically and efficiently lower gene expression from a donor carrying over 125 CAG repeats.

Lowering expression of each member of the MMR complexes MutS (MSH2, MSH3, and MSH6), MutL (MLH1, PMS1, PMS2, and MLH3), and LIG1 resulted in characteristic MMR deficiencies. Reduced MSH2, MSH3, and MLH1 slowed repeat expansion to the largest degree, while lowering either PMS1, PMS2, or MLH3 slowed it to a lesser degree. These effects were recapitulated in iPSC-derived striatal cultures where MutL factor expression was lowered.

CRISPRi-mediated lowering of key MMR factor expression to levels feasibly achievable by current therapeutic approaches was able to effectively slow the expansion of the HTT CAG tract. We highlight members of the MutL family as potential targets to slow pathogenic repeat expansion with the aim to delay onset and progression of HD and potentially other repeat expansion disorders exhibiting somatic instability.

Introduction

Huntington disease (HD [MIM: 143100]) is a dominantly inherited fully penetrant neurodegenerative disease characterized by motor, cognitive, and psychiatric symptoms with progression to death typically 15–20 years from onset. The mean age of onset is ~43 years old, and we currently have no disease-modifying therapeutics. The onset and neurodegenerative pathophysiology of HD are driven by the inexorable increase in length of the expanded CAG repeat tract in exon 1 of the *huntingtin* gene (*HTT* [MIM: 613004]) throughout the lifetime of the individuals carrying the pathogenic allele.[1–5] Here, we examine candidate HD modifier genes identified through genetic studies to validate targets to pursue in slowing repeat expansion rates, with the hope to delay disease onset and slow progression.

Expansion of the CAG repeat beyond a pathological threshold leads to progressive neurodegeneration.[6] Striatal medium spiny neurons (MSNs) show particular vulnerability, and degeneration is detectable well before onset.[7–12] Striatal MSNs and layer V cortical pyramidal cells more frequently carry high repeat length burdens than other neuron sub-types in both mouse and human,[8,10,13,14] suggesting that increased repeat expansion is a significant driver of vulnerability in these neurons. Although CAG repeat length correlates with its expansion rate, additional cell-type-dependent modifiers that drive somatic CAG repeat mosaicism within an individual are not understood.[14–16]

Increasing length of the *HTT* CAG repeat robustly correlates with earlier age at onset (AaO) and disease progression; however, substantial variation exists between HD carriers with similar repeat lengths.[1,2,17]. Genome-wide association studies (GWASs) have identified variants associated with altered AaO and progression in HD cohorts controlled for repeat length.[1,2,18–20] Many variants are located in DNA damage response (DDR) genes. Variants in the Fanconi-anaemia pathway-associated protein FAN1 (MIM: 613534) strongly associated with altered AaO, and FAN1 has been convincingly shown to significantly alter repeat expansion in multiple models.[21–23] Disease-modifying variants in multiple members of the mismatch repair (MMR) pathway were also identified, including MSH3 (MIM: 604395), MLH1 (MIM: 120436), PMS1 (MIM: 600258), PMS2 (MIM: 600259), and LIG1 (MIM: 126391).

MMR has long been implicated in instability at the *HTT* CAG repeat.[24] Effective MMR in mammalian cells relies on the MutS heterodimer complexes—either MSH2 (MIM: 609309) and MSH6 (MIM: 600678) (MutSα) or MSH2 and MSH3 (MutSβ). While MutSα primarily engages 1–2 base mismatches, MutSβ recognizes larger extra-helical

[1]Huntington's Disease Centre, Department of Neurodegenerative Disease, UCL Queen Square Institute of Neurology, University College London, London WC1N 3BG, UK; [2]Dementia Research Institute at UCL, London WC1N 3BG, UK
*Correspondence: s.tabrizi@ucl.ac.uk
https://doi.org/10.1016/j.ajhg.2024.04.015.
© 2024 The Author(s). This is an open access article under the CC BY license (http://creativecommons.org/licenses/by/4.0/).

extrusions, though with some overlap.[25,26] *In vitro* studies propose that MutSβ associates with extra-helical DNA structures similar to those that occur at highly repetitive sequences like the *HTT* CAG repeat.[27] Engagement of a lesion by either MutS complex recruits MutL protein heterodimers to form ternary complexes. MLH1 serves as the common component in each MutL heterodimer, partnering with either PMS2 (MutLα), PMS1 (MutLβ), or MLH3 (MIM: 604395; MutLγ).[28] Upon activation by MutS and PCNA (MIM: 176740), MutLα incises DNA flanking the mismatch,[29] while MutLγ nicks in the opposing strand.[30] Unlike PMS2 and MLH3, PMS1 lacks a nuclease domain, so MutLβ function in MMR is unknown. At conventional MMR substrates, exonuclease EXO1 resects the lesion-containing strand, which then undergoes resynthesis and ligation facilitated by DNA polymerase δ and LIG1.[28]

Substrate, kinetics, ternary complex formation, and the co-factors present at unstable repeats are not well understood in comparison to non-repeat MMR lesions, yet additional repeats are inevitably incorporated. Loss of individual MMR proteins in mouse models significantly affects repeat expansion: loss of MSH3 arrests repeat expansion in HD and in myotonic dystrophy (MIM: 160900) mouse and cell models,[22,24,31–35] while MSH6 loss has minor variable effects.[36,37] Likewise, loss of MLH1, PMS1, and MLH3 slows repeat expansion in HD and Friedrich's ataxia (FRDA [MIM: 229300]) models,[38,39] while PMS2 both promotes and suppresses expansion in different models.[38,40–42]

The process of somatic repeat expansion in the brain appears to be a major factor in driving disease progression, leading to production of toxic HTT protein species. Rather than a continuum of repeat length-dependent toxicity, it has been suggested that there is a critical threshold beyond which severe dysfunction occurs in vulnerable cells.[43] Slowing the rate of expansion is clearly an attractive target for therapeutic intervention in HD and other repeat expansion disorders that exhibit somatic instability, but first it is necessary to confirm which disease-modifying candidates actually play a role in the expansion process. The occurrence of gene function-modifying variants within the population, as identified by GWASs, suggests their effects are tolerated to some degree. Therefore, targeted expression modulation of these genes could also likely be tolerated in individuals. For example, functionally deleterious polymorphisms in *MSH3* slow repeat expansion and HD progression,[44] and lowering MSH3 expression is currently being pursued therapeutically.[45]

Here, we have asked whether lowering expression of candidate MMR genes to levels achievable by current therapeutics can affect the rate of repeat expansion in a human *ex vivo* model of HD. We investigated HD modifier MMR genes and their cognate heterodimer partners by targeting MutS (MSH2, MSH3, and MSH6), MutL (MLH1, PMS1, PMS2, and MLH3), and LIG1 expression using a CRISPR interference system in HD induced pluripotent stem cells (iPSCs). This lowers expression by targeting a catalytically inactive Cas9 fused to a transcriptional repressor to the transcriptional start site of each gene. The effects of lowering each target on repeat expansion were then assayed both in dividing HD iPSCs and HD iPSC-derived post-mitotic striatal neurons.

Material and methods

Cell culture

QS5.1 HD 125Q iPSCs ("125Q iPSCs") were derived previously from fibroblasts from a 7-year-old female pediatric HD individual with fully informed parental consent and ethical approval from the local ethics committee with good clinical practice (GCP) compliance.[22] The fibroblasts carried 125 uninterrupted HTT CAG repeats and were reprogrammed by Sendai-based methods at Censo Biotechnologies. iPSCs were maintained in E8 Flex (Gibco) on Geltrex (1:100, Gibco)-coated plasticware (Nunc) and passaged using 0.5 mM EDTA in PBS (Gibco). U2OS cells were maintained in Dulbecco's modified Eagle medium (DMEM) supplemented with 10% FBS, 1% L-glutamine, and 1% pen/strep (Gibco) and passaged using Trypsin/EDTA.

U2OS repeat expansion assay

U2OS cells were transduced with p'HRsincpptUCOE HTT Ex1 118Q IRES eGFP and cultured for the indicated time periods and changes in CAG repeat length determined by fragment analysis, as previously reported.[21]

Striatal differentiation of iPSCs

Differentiation was performed based on Arber et al.[46] iPSCs were passaged 1:2 onto Matrigel-coated plates to become confluent within 24–48 h with feeding every 24 h. Once confluent, cells were washed and switched to neural induction media (DMEM:F12 and Neurobasal A 1:1 [both Gibco] with 1× L-glutamine [Gibco], 1× PenStrep [Gibco], 0.5× N2 [Gibco], 0.5× B27 without vitamin A [Gibco], 100 μM β-mercaptoethanol with 100 nM LDN-193189 [Sigma], and 10 μM SB431542 [Cambridge Biosciences]). Media was changed every 24 h up to day 10 on which cells were mechanically clump passaged using 0.02% EDTA in PBS onto fibronectin-coated plates and maintained in the same media without mothers against decapentaplegic homolog (SMAD) inhibitors and including 25 ng/ml activin-A (Peprotech). All subsequent media changes were half volumes and every 48 h before passaging again onto poly-*l*-ornithine and laminin-coated 12-well plates on day 20. After day 26, B27 was switched to include vitamin A and both brain-derived neurotrophic factor (BDNF) and glial cell line-derived neurotrophic factor (GDNF) included at 10 ng/ml (Peprotech). Activin-A was omitted from day 36 with half media changes every 72 h.

Design, cloning, and preparation of CRISPRi guides

CRISPRi single guide RNAs (sgRNAs) for knocking down MMR component expression were designed and compared using the online tools CRISPick (portals.broadinstitute.org/gppx/crispick/) and CRISP-ERA (crispr-era.stanford.edu/) (Table S1). Non-targeting control guide sequences were obtained from published guide libraries.[47] Corresponding oligo pairs were hybridized and inserted into the pLentiGuide vectors by GoldenGate cloning using BsmBI-V2 and T4 Ligase (NEB) and then transformed in NEB-stable cells. Isolated clones were screened by colony PCR across the stuffer insert and confirmed by restriction digest followed by sequencing from the U6 promoter. Primer sequences can be found in Table S1.

Endotoxin-free plasmid for transfection was purified using PureYield column kits (Mini/Maxi, Promega). Plasmid DNA for direct use in electroporation of iPSCs was further concentrated by precipitation. Plasmid DNA used to generate lentiviral particles were transfected into HEK293 cells using LentiX single-shot VSV-G (Takara) following manufacturer's instructions. Harvested virus was concentrated by centrifugation through a 10% sucrose cushion at 10,000 g for 4 h.[48] Viral titer was determined by qPCR (LentiX, Takara).

Modification of guide and dCas9-KRAB constructs

The KOX1 KRAB domain of the dCas9-KRAB fusion in the CLYBL-targeting vector pC13 dCas9-TagBFP-KRAB (addgene #127968[49]) was swapped for ZIM3 KRAB domain from pLX303-ZIM3-KRAB-dCas9 (addgene #331490[50]). The ZIM3 coding sequence was amplified by PCR using a pLX303 as template. A 5′ HindIII site and a 3′ FseI site were appended by inclusion in the primer sequences. The KRAB domain in the dCas9-tagBFP-KRAB degron construct pRT029 was dropped out by HindIII/FseI digest and replaced by the HindIII/FseI-digested ZIM3 amplicon. ZIM3 and TagBFP were amplified with overlapping homology with the pC13 5′ BamHI and 3′ MluI sites. The final pC13 dCas9-BFP-Zim3 construct was assembled from these fragments using HiFi assembly mix (NEB).

pLentiGuide-Hygro-eGFP (addgene #99375[51]) was modified to include PiggyBAC inverted terminal repeats flanking the sgRNA and HygroR-eGFP expression cassette. A backbone fragment containing AmpR was excised by FspI. The same fragment was amplified by PCR using primers appended with the PiggyBac terminal inverted repeats (PB TIR) sequences and blunt cloned back into the same sites.

All restriction enzymes were purchased from NEB. All PCR-based cloning steps were performed using KAPA HiFi taq (Roche) and verified by sequencing; primer sequences can be found in Table S2. An expression vector for the hyperactive PiggyBAC transposase with an mCherry reporter (pRP EF1A-hyPBase CMV-mCherry) was synthesized by Vectorbuilder.

TALEN-mediated knockin of dCas9-ZIM3 in 125Q HD iPSCs

The modified pC13 dCas9-BFP-ZIM3 construct with CLYBL homology arms was co-nucleofected into 125Q iPSCs with 2 additional constructs carrying transcription activator-like effector nucleases (TALENs) targeting the CLYBL locus (pZT-C13-R1 and pZT-C13-L1, addgene #62196, #62197[52]) in P3 solution using program CA137 in a 4D-Nucleofector (Lonza). Cells were seeded at clonal density, and selection in G418 was initiated 48 h later. Colonies were picked and screened for blue fluorescent protein (BFP) expression. PCRs were performed spanning both the 3′ and 5′ homology arm junctions to identify integration at the intended sites.

Zygosity was determined by qPCR using genomic DNA as template using primers for Cas9 and ZNF80. A two-copy reference was created by cloning the ZNF80 amplicon into the AleI site in pLX303. Copy-number qPCR was performed using Forget-Me-Not qPCR Master Mix (Biotium) on a QuantStudio5 system.

gRNA introduction by lentiviral transduction

iPSCs were transduced at 70% confluence with ~50 MOI in the presence of 10 μg/ml polybrene in 24-well plates. After 24–48 h, cells were passaged to 6-well plates and selection was initiated in 50 μg/ml hygromycin. Selection was maintained for 2 weeks before assaying knockdown.

gRNA introduction by PiggyBAC transposition

iPSCs at 70% confluence were preincubated for 30 min with ROCKi (10μM Y-27632, Sigma) and then dissociated to single cells using TrypLE (Gibco). Cells were resuspended in complete media with ROCKi, centrifuged at 250 g for 3 min, and then resuspended in PBS and counted. 2×10^5 cells/ml were nucleofected in 20 μL using nucleocuvette strips with 0.5 μg guide plasmid, 0.5 μg hyPBase plasmid, and 0.1 μg BCL-XL plasmid.[53] After 24–48 h, cells were passaged to 6-well plates, and selection was initiated in 50 μg/ml hygromycin. Selection was maintained for 2 weeks before assaying knockdown.

Generation of CRISPR-Cas9 knockout cell lines

MSH3 and MLH1 were knocked out in U2OS cells previously.[22] MSH6 knockout U2OS and 125Q iPSCs were generated by nucleofection with complexed HiFi Cas9 and paired Alt-R guide RNAs (Integrated DNA Technologies, IDT) targeting exon 2 or exon 4 (Table S3). For both methodologies, single cell clones were isolated, expanded, and screened by PCR for the deletion of the guide flanked sequence (Table S2), and then knockout was confirmed by Sanger sequencing and western blot.

RT-qPCR

Total RNA was prepared by Qiagen RNAeasy columns from cells lysed directly in wells in RLT buffer. cDNA synthesis was performed using the SuperScript IV First-Strand Synthesis System with 1:1 Oligo dT and random hexamer primers (all Thermo). qPCRs were set up using 10 ng template in 15 μl reactions with TaqMan Fast Advanced mastermix (Thermo) with assays in two-plex (Table S4) on a Quantstudio 5. All qPCRs were performed in technical triplicate using exon-spanning assays and presented as fold change relative to the geometric mean of three references genes (UBC, ATP5B, EIF4A2).

Western blot

Frozen cell pellets were lysed in 2 pellet volumes of RIPA (Sigma) with protease inhibitors (Halt, Promega) by trituration followed by a 20-min incubation on ice with Benzonase (Sigma). Protein concentration was determined by BCA assay (Pierce), and 20 μg of protein were run on 4%–12% Bis-Tris NuPage gels (Thermo) against Spectra Multicolour high-range markers (Thermo) before transfer to 0.2 μm nitrocellulose overnight using an XCell ii system at 35 V (Thermo). Membranes were blocked in 5% milk in PBS for 1 h before incubating with primary antibodies overnight at 4°C with shaking. After 4 washes of 10 min each with PBS with 0.1% Tween 20 (PBST), membranes were incubated with secondary antibodies for 1 h at room temperature. After 4 further washes with PBST and 2 with PBS, membranes were imaged on a Licor Odyssey CLx. Band intensity of proteins of interest are presented relative to β-actin (ACTB) and normalized to non-targeting controls. Antibodies and dilutions can be found in Table S5.

MMR-deficiency assay

Method adapted from Gupta et al.[54] iPSCs were pretreated for 2 h in StemFlex media (Gibco) with 1× Revitacell (RC; Gibco) and 25 μM O6-Benzylguanine (O6BG; Cayman). Serial dilutions of N-methyl-N′-nitro-N-nitrosoguanidine (MNNG; Selleckchem) in StemFlex with RC and O6BG were preplated on geltrex-coated (Gibco) 12-well plates. iPSCs were dissociated to single cells using Accutase (Invitrogen). Suspension was washed in PBS, resuspended in

StemFlex +RC and BG, and passed through a 30-micron cell strainer (MACS). Cell density and viability was counted in trypan blue on a Tecan cell chip. 2,000 cells were seeded into the prepared plates. Media was changed after 16 h, and colonies were allowed to grow for 7 days. The same protocol was used for 6-thioguanine treatment, omitting O6BG and using E8 Flex and 10 μM Y-27632 in place of StemFlex and RevitaCell, respectively. Plates were fixed for 10 min in 4% paraformaldehyde (PFA) at RT and washed with PBS. Colonies were stained with 0.01% crystal violet (Gurr) in 10% ethanol for 10 min and washed extensively. Colonies were counted in ImageJ. Data from 3 experiments in triplicate with colony frequency normalized to untreated of the same genotype. Normality was determined using the D'Agostino-Pearson test followed by ANOVA and Bonferroni post hoc in Prism (GraphPad).

Immunocytochemistry

Cells cultured in 96-well PhenoPlates (PerkinElmer) were fixed in 4% PFA in PBS for 15 min at RT, followed by 3 PBS washes of 10 min each. Fixed cells were incubated in blocking buffer comprised of 5% BSA (Sigma), 1% FBS (Gibco), and 0.5% Triton X-100 (BDH) in PBS. Samples were incubated with primary antibodies overnight in diluent (blocking buffer 1:10 with PBS). After 4 washes of 10 min with PBST, samples were incubated with secondary antibodies and Hoechst counterstain for 2 h RT. Following 4 further 10-min PBST washes and 2 PBS washes, samples were prepared for microscopy with 90% glycerol and 0.5% N-propyl gallate in 0.1 M Tris pH 7.4. Images were acquired on an Opera Phenix (PerkinElmer) from at least 4 random fields in 3 wells from each experiment. Images for quantification were acquired with identical setting with flat-field correction and further analyzed using Fiji.[55] Normality was determined using the D'Agostino-Pearson test and tested by ANOVA and Bonferroni post hoc in Prism 9 (GraphPad).

Fragment analysis

DNA was prepared by either QuickExtract (Lucigen) from iPSCs or Qiagen DNeasy columns (MSNs and U2OS) following manufacturer's instructions. PCR across the *HTT* CAG repeat was performed on 10 ng gDNA using AmpliTaq Gold 360 (Thermo) with 6-FAM labeled HD3F CCTTCGAGTCCCTCAAGTCCTT[56] and HD5 Mangiarini CGGCTGAGGCAGCAGCGGCTGT,[57] with the following cycling conditions: 94°C for 90 seconds, 35 cycles of 94°C for 30 seconds, 65°C for 30 seconds, and 72°C for 90 seconds, and the final extension at 72°C for 10 min 2 μL of PCR product was denatured in 10 μL HiDi formamide (Thermo) with 0.5 μL Mapmarker ROX 1000 (Eurogentec) and run on an ABI 3730XL genetic analyzer. Sequencer output was processed using GeneMapper (Thermo) and a custom R script is available at https://michaelflower.org using a threshold of 20% of modal peak height and a correction factor of 2.724117. Data presented as modal repeat length relative to control samples and a modified instability index,[32] calculated here as the sum of the change in repeat length relative to the mode, multiplied by proportional peak height, for each peak in the distribution but measured relative to the control repeat length. Data were analyzed by linear mixed-effects model with post hoc comparison by Tukey's method in Prism 9 (GraphPad).

Genetic constraint in selected HD modifier and DNA repair genes

To measure genetic constraint, or the gene's intolerance to variation, we analyzed predicted loss-of-function (pLoF) metrics from the gnomAD database (v.4.0.0; 807,162 samples, https://gnomad.broadinstitute.org/).[58] This analysis calculated the ratio of observed/expected (o/e) number of LoF variants using transcripts annotated by GENCODE v39 and the GRCh38 reference. The expected counts are based on a model that accounts for sequence context and methylation. pLoF values are presented along with the 90% confidence interval. When a gene has a low pLoF value, it is under stronger selection. For example, a pLoF value of 0.2 means that there were 20% of the expected number of variants observed in that gene. While pLoF values are continuous, scores below 0.6 are generally accepted to indicate significant selection against LoF variation.

Results

Construction of a stable CRISPRi HD iPSC line

We constructed a CRISPRi HD iPSC line in which we could both effectively lower target gene expression and measure instability at the *HTT* CAG repeat. A dCas9-KRAB repressor construct was stably integrated into the CLYBL safe harbor in chromosome 13 of our highly characterized 125Q juvenile HD iPSC line.[22] We modified the CLYBL targeting construct of Tian et al.[49] to swap the KOX1 KRAB domain for the more effective ZIM3 KRAB domain identified by Alerasool et al.[50] (Figure 1A). We compared these fusions using *PMS2* targeting guides and found ZIM3-mediated repression was equal to or greater than that observed with the same guide sequences paired with the KOX1 KRAB fusion (Figure S1A).

Targeted integration of the construct at the CLYBL locus was directed by TALEN-mediated double-strand breaks and subsequent homology-directed repair using the homology arms in the dCas9-repressor donor construct (Figure 1A). TagBFP expression was used to screen single-cell iPSC clones for successful knockin after selection in G418 (Figure 1B). We further screened 6 of these clones by PCR to amplify an internal Cas9 product, a product spanning the 5′ and 3′ junction between the CLYBL locus and the donor construct, and a product spanning the TALEN targeted cut site (5′–3′ spanning, Figure 1C). These PCRs confirmed knockin at the correct site in all clones and indicated 3 of the 6 clones were homozygous for the CRISPRi construct. This was further confirmed by copy-number qPCR (Figure 1D), which also highlighted 1 of the 3 potential heterozygous clones as potentially mixed/non-clonal. The range of modal CAG length of the knockin clones remained within ∼20% of parent population (Figure 1E). Clones maintained good iPSC-like morphology, growth, and pluripotency-associated marker expression (Figures 1B and 1F). *OCT3/4* and *NANOG* transcript levels were not significantly different from those of the parent 125Q line by RT-qPCR (Figure 1F). Immunostaining for the pluripotency markers OCT4, NANOG, LIN28, and SSEA4 showed expected distributions and levels comparable to that of the parent line (Figure 1B). TagBFP distribution showed that the dCas9-TagBFP-ZIM3 fusion protein was localized to the nucleus as expected

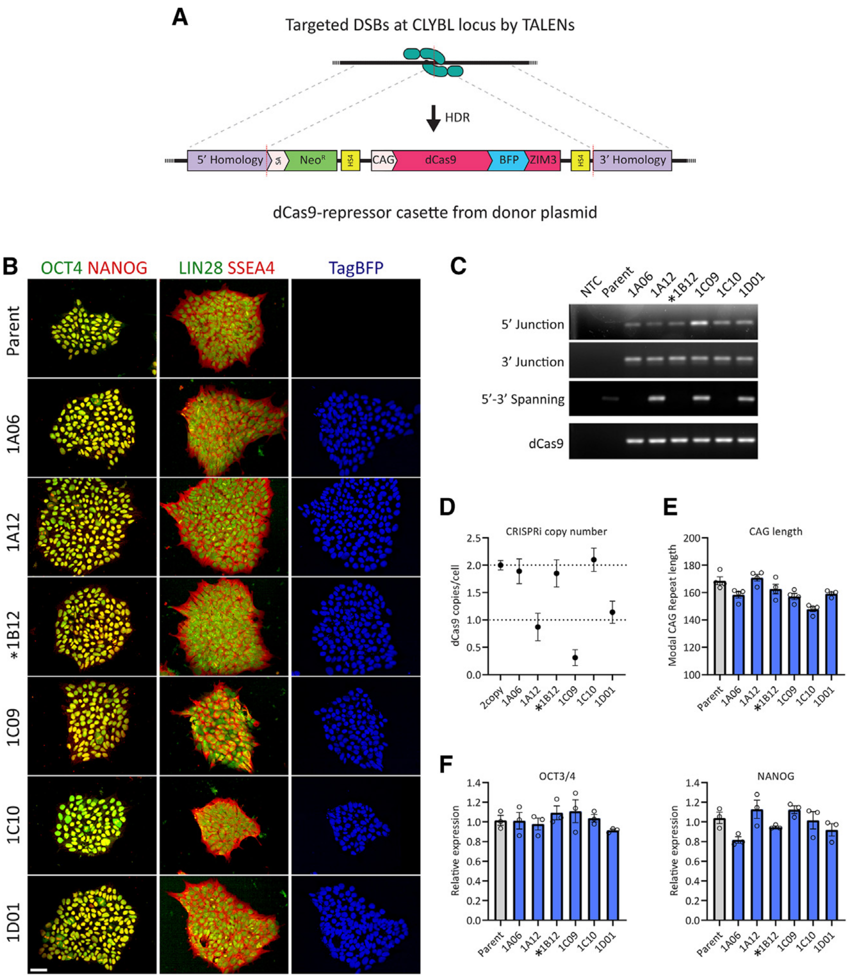

Figure 1. Construction and validation of the 125Q HD CRISPRi iPSC lines
(A) Schematic showing TALEN-mediated knockin of a dCas9-tagBFP ZIM3 KRAB construct into the CLYBL safe harbor.
(B) Single-cell clones were isolated and screened for TagBFP expression and pluripotency-associated marker expression. Scale bars, 50 μm.
(C) Accurate knockin was verified by PCR across homology arm junctions (5′ and 3′ junctions) and insert site spanning (5′ to 3′ spanning) PCR alongside an internal dCas9 PCR.
(D) Zygosity and off-target integration was assessed by copy-number qPCR.
(E) Modal CAG length was determined by fragment analysis.
(F) Expression of pluripotency-associated genes was further assessed by qPCR for OCT3/4 and NANOG relative to the parental iPSC line.
Clone 1B12 (*) used for further work.
Mean values ± SEM in triplicate.

(Figure 1B). Clone 1B12 was used as the parent line for further CRISPRi work.

Identification of guide sequences that effectively lower MMR factor expression

Multiple guide sequences were selected that target the transcriptional start site of each gene of interest: *MSH2*, *MSH3*, and *MSH6* (MutS components); *MLH1*, *PMS1*, *PMS2*, and *MLH3* (MutL components); and *LIG1* (Figure 2A). Oligos carrying these guide sequences were cloned into pLentiGuide-based expression constructs (Figure S1B). These constructs also carry EGFP and hygromycin resistance markers and were introduced to the CRISPRi HD iPSC line either by lentiviral transduction (MutL) or PiggyBAC transposition (MutS and LIG1) (Figure 2B). Both methods resulted in comparable initial efficiencies by EGFP expression with ~20%–30% GFP^+ cells at 24 h post-introduction.

Up to 5 guides per target were introduced in parallel alongside non-targeting guides (NTC1 and 2) (Figure 2C). After selection and expansion in hygromycin-containing medium, target expression was assayed at the transcript level by qPCR. Significantly lower target transcript levels were seen with each guide in comparison to the conditions without guides or with non-targeting guides. Lowering of transcript levels to around 70% was seen for the top MSH2 and MSH3 guides, while MSH6 and LIG1 guides achieved around 60%–70% lowering (Figure 2D). At least 2 guides for each MutL component (MLH1, PMS1, PMS2, or MLH3) lowered transcript levels by around 80% (Figure 2E). From these sets, 2 guides with good efficacy were selected to characterize the consequences of lowering each target on CAG repeat expansion (Figures 2D and 2E, dashed boxes). First, however, they were characterized in terms of effect on target protein levels and MMR proficiency.

CRISPRi-mediated lowering of MutS and MutL factors in iPSCs leads to characteristic MMR deficiencies

Homogeneous expression of EGFP from the guide construct can be seen across the non-targeting and targeting guides after selection (Figure 3A). Immunocytochemistry for each CRISPRi MMR protein target shows a primarily nuclear distribution in iPSCs, with some variation in intensity likely linked to cell-cycle status.[59] This pattern remains where target transcript levels are lowered by CRISPRi, though at substantially reduced intensity (Figure 3A).

Quantification of the median nuclear intensity of each target within MutS and LIG1 (Figure 3B) and MutL (Figure 3C) confirms the homogeneity of the knockdown and correlates with the reduction in transcript level seen for each guide (Figures 2E and 2F, dashed boxes). Significant and consistent lowering is seen between cells within each knockdown population.

Total loss, reduced levels, or functional mutations in the various members of the MMR pathway leads to characteristic defects in MMR response.[60] We have assayed MMR

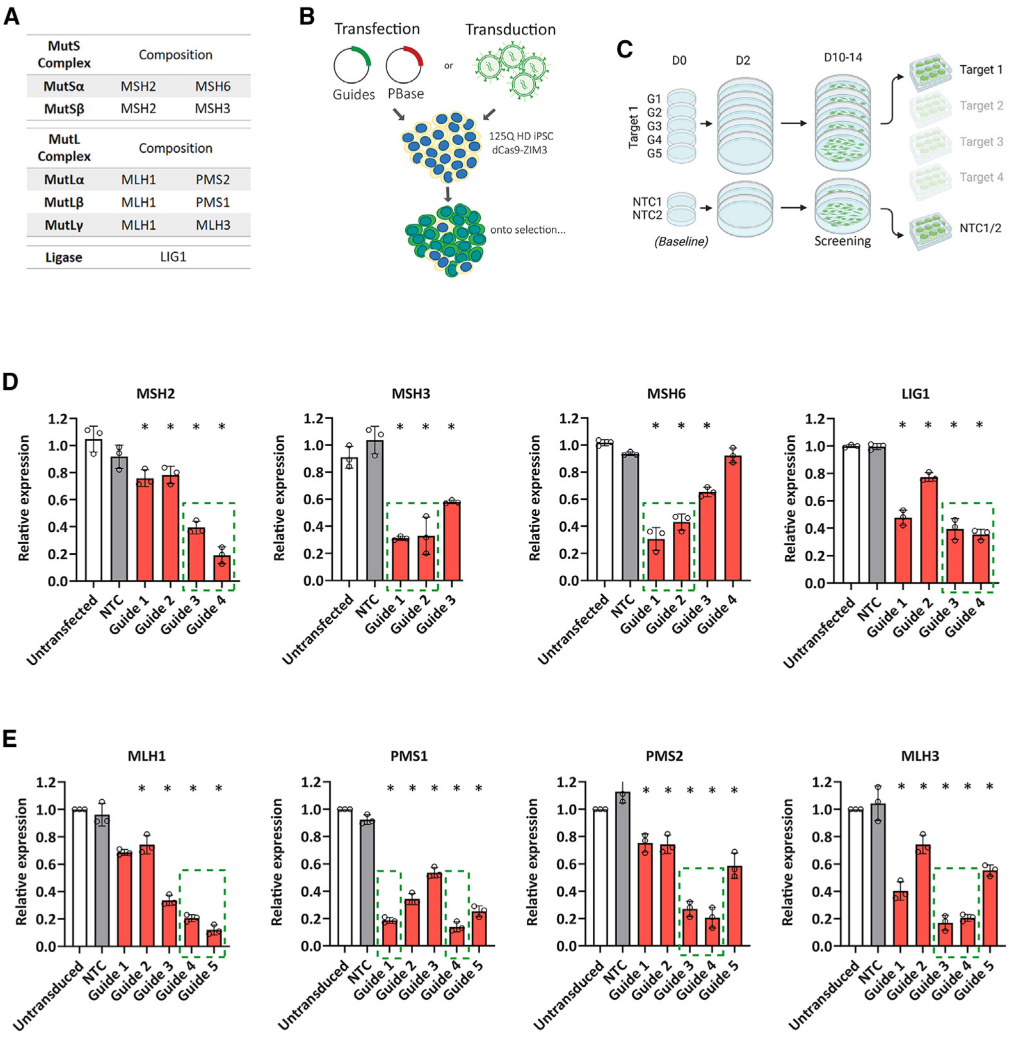

Figure 2. Effective knockdown of MMR factors after introduction of sgRNAs to the HD CRISPRi line

(A) Guide sequences were introduced into the dCas9-repressor iPSC line to target MMR gene expression.

(B) Guides targeting the MutL factors MLH1, PMS1, PMS2, and MLH3 were introduced by lentivirus, while MutS factors MSH2, MSH3, and MSH6 as well as LIG1 were introduced by electroporation and PiggyBAC transposition.

(C) Up to five guides per target and two non-targeting (NTC) guides were used in each round, and their efficacy in lowering target expression was assayed after selection in hygromycin.

(D and E) Transcript levels were determined by qPCR for MutS and LIG1 (D), and MutL (E) normalized to un-transfected/transduced controls.

Data compared by ANOVA with Bonferroni's post hoc. Error bars show SEM. $N = 3$, error bars $\pm$SEM, $* = p < 0.05$. Two guides per target were taken forward, highlighted by green dashed boxes.

proficiency here using survival after treatment with the alkylating agent MNNG.[61] Alkylation of a DNA base results in a lesion mimicking a mismatched base pair during DNA synthesis. This leads to MMR-dependent cell-cycle arrest, futile repair attempts, and cell death, whereas in the absence of MMR, cells survive.[62] Non-targeting control iPSCs and those with CRISPRi-lowered MMR factor expression were seeded at clonal density and treated with a range of MNNG concentrations for 16 h followed by quantification of the frequency of colonies formed after 1 week.

The small single-base lesions associated with MNNG methylation would be repaired primarily through MutSα (MSH2:MSH6)-dependent means.[61] As expected, the loss of the core MutS and MutL factors had the largest effect on survival, as did lowering MSH6 (Figures 3D and 3E, $p < 0.05$). In contrast, lowering MSH3 or LIG1 had no significant effect on MNNG sensitivity in comparison to controls. Lowering the MutL co-factors PMS2 and MLH3 had an intermediate effect in comparison to lowering their partner MLH1 ($p < 0.05$), while lowering PMS1 showed no significant difference in survival to controls (Figures 3D and 3E).

CRISPR-Cas9-mediated knockout of MSH6, MSH3, and MLH1 in U2OS osteosarcoma cells resulted in the complete loss of each protein (Figure S2A[22]). MMR proficiency in these cells was assayed using the base analog 6-thioguanine (6TG), which also results in unrepairable mismatch-like DNA lesions.[63] Both MSH6 and MLH1 null U2OS cells

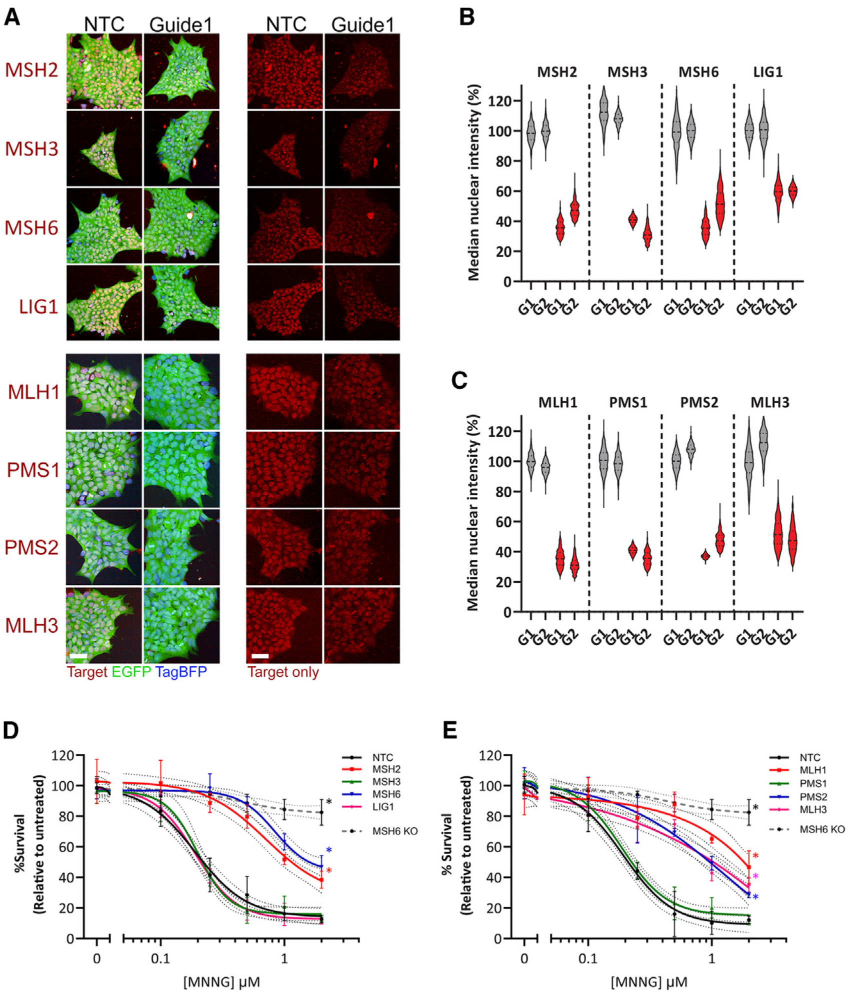

Figure 3. Homogeneous lowering of MMR factor expression in CRISPRi pools leads to characteristic MMR deficiencies

(A) Immunostaining for indicated target proteins in red channel shows knockdown is consistent cell-to-cell.

Representative images for a non-targeting control guide and a targeting guide. EGFP from the guide vectors and TagBFP from the dCas9-KRAB knock-in. Scale bars, 50 μm.

(B and C) Quantification of fluorescence intensity for each target normalized to NTC1 levels for MutS and LIG1 (B) and MutL (C). Line indicate median values, 1–2,000 cells per plot from four random positions in three independent experiments.

(D and E) iPSC colony formation after treatment with the indicated concentration of methylnitronitrosoguanidine (MNNG) for CRISPRi lowered MutS and LIG1 (D) and MutL (E).

Mean colony frequency ± SEM in triplicate as a proportion of untreated cells of the same genotype, normalized to non-targeting control iPSCs (NTC) and shown alongside MSH6 knockout (MSH6 KO) iPSC survival. Dotted lines 95% CIs, *$p < 0.05$.

showed improved survival post-6TG treatment, in contrast to wild-type and MSH3 null cells (Figure S2B). Similarly, the same MSH6 knockouts made in the 125Q iPSC line (Figure S2C) resulted in the most robust survival post-6TG or MNNG treatment, indicating that the effect seen upon lowering by CRISPRi in iPSCs likely does not represent the maximal MMR deficiency possible in each case (Figures 3D, 3E, and S2D–S2F).

The lack of MMR deficiency seen where LIG1 has been lowered may suggest either further lowering is required or there is redundancy from other LIG family members, such as LIG4, whose levels remain unaffected (Figure S3). Having shown that lowering MutS or MutL levels to the degree achieved here is sufficient to detect an MMR phenotype, we went on to characterize their effect on CAG repeat expansion in dividing iPSCs and post-mitotic MSNs.

Lowering MutSα and each MutL component slows repeat expansion in dividing iPSCs

We measured the effect of target lowering on repeat instability in iPSCs over the course of 80 days during routine passaging. CAG repeat length was measured by fragment analysis at 20-day intervals, and changes in modal CAG length or instability index[32] were reported relative to the baseline at the start of the time course (Figure 4A; representative traces presented in Figures S4A and S4B). No substantial changes in target transcript levels were observed across the time course by qPCR, indicating that no significant silencing of any CRISPRi component was occurring (Figure 4B).

Over the 80-day time course, we saw an increase in modal CAG length of 4–6 from baseline (Figures S4A and S5A) and an increase in instability index by around 2 units in NTC guide cultures (Figure 4C). Across the same period, an increase of less than 1 unit was measured in the instability index of cultures where expression of either MSH2 or MSH3 was lowered (Figure 4C, $p < 0.005$), accompanied by a modal increase of only 1–2 CAG units ($p < 0.005$, Figure S4A). In contrast, no significant change in modal CAG repeat length or instability index was found between control cultures and those with lowered MSH6 or LIG1 (Figures 4C and S4A). Where MutL expression was targeted (Figure 4D), we observed the slowest rates of expansion where MLH1 levels were lowered, with a change in instability index relative to baseline around 1 unit ($p < 0.005$) and an increase of only ~1 CAG by day 80 ($p < 0.005$, Figures S4B and S5A). Lowering PMS1, PMS2, and MLH3 also had a small but significant effect, reducing instability index by around half a unit and modal repeat length by ~1–2 CAGs over the full time course in comparison to control cultures ($p < 0.05$, Figures 4D and S4A). Rates of change in instability index and modal CAG per day were derived from the mixed models of the 4 independently passaged

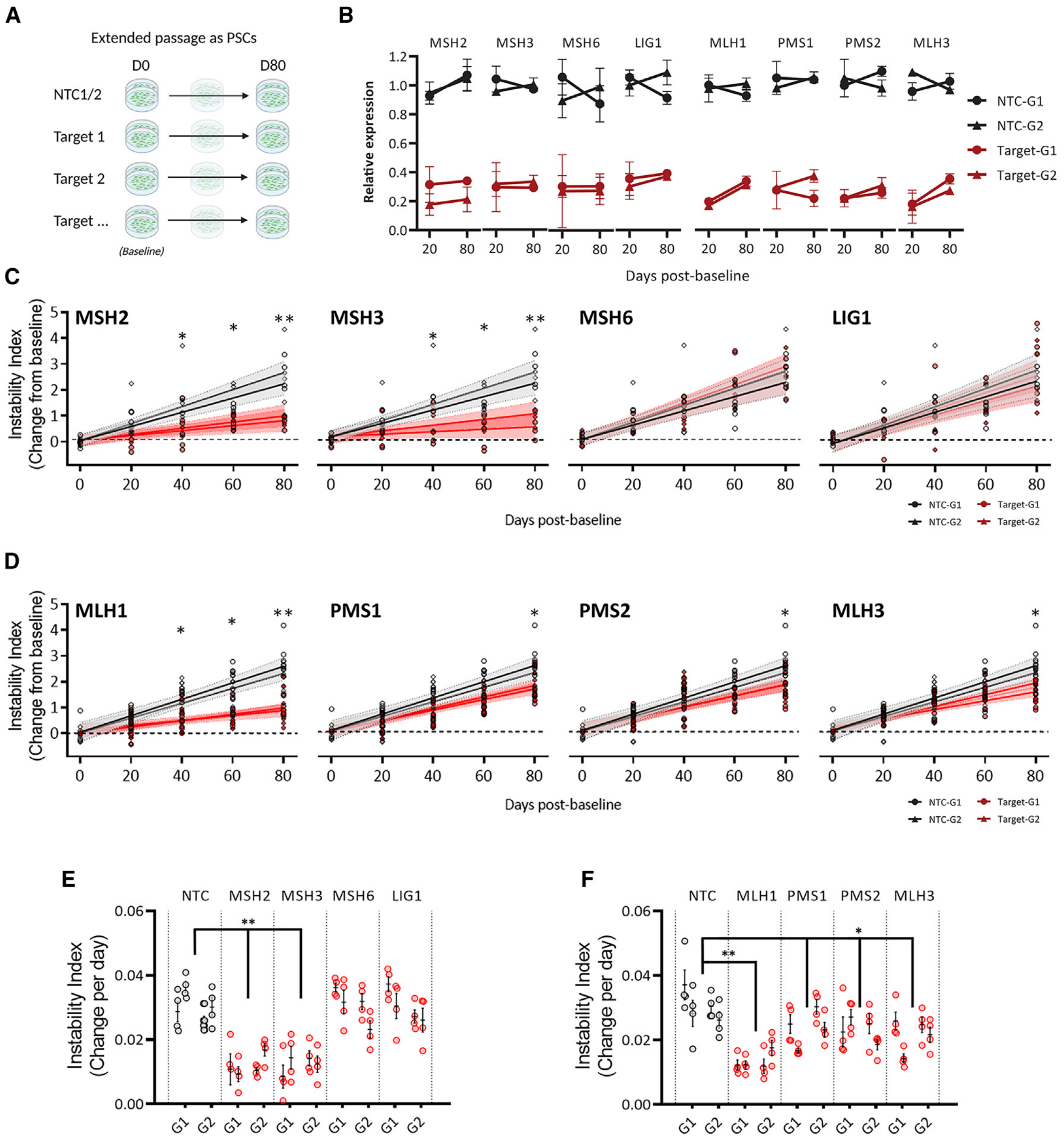

Figure 4. Reduced expression of MutS and MutL components slows repeat expansion in dividing iPSCs
(A) Repeat instability at the pathogenic CAG repeat tract in the 125Q HD iPSC line can be measured over time in across routinely passaged cultures, relative to the starting point at day 0 (D0, baseline).
(B) qPCR for each target shows lowering persists over the time course.
Mean expression relative to day 0 ± SEM in triplicate for two NTC guides and the selected two target-specific guides at day 20 and 80.
(C and D) Instability index relative to control over an 80-day period for MutS and LIG1 (C), and MutL (D) lowered cultures relative to baseline on D0. Four cultures per guide passaged in parallel each from two independent CRISPRi pools, dashed lines 95% confidence intervals.
(E and F) Rate of change in instability index relative to control per day for MutS and LIG1 (E) and MutL (F). Representative fragment analysis traces can be found in Figure S4.
Open circles represent parallel cultures with mean bar ±SEM. *$p < 0.05$ **$p < 0.005$.

cultures per guide and transduction. Lowering MSH2, MSH3, and MLH1 reduced the rate of expansion in iPSCs by between 60% and 65% (Figures 4E and 4F), while lowering PMS1, PMS2, or MLH3 reduced the rate by between 25% and 35% compared to control rates (Figure 4F).

These results matched data obtained using a second model of repeat expansion in U2OS cells. In these cells, repeat expansion can be measured over time in a 118Q repeat present in a *HTT* exon1 expression construct delivered by transduction.[21,22] The effect on repeat expansion in this construct was measured in MSH3, MSH6, and MLH1 knockout cells over a 40-day period (Figure S6). As expected, the total absence of either MSH3 or MLH1 resulted in an almost complete arrest of expansion, while loss of MSH6 had no significant effect. Rescuing

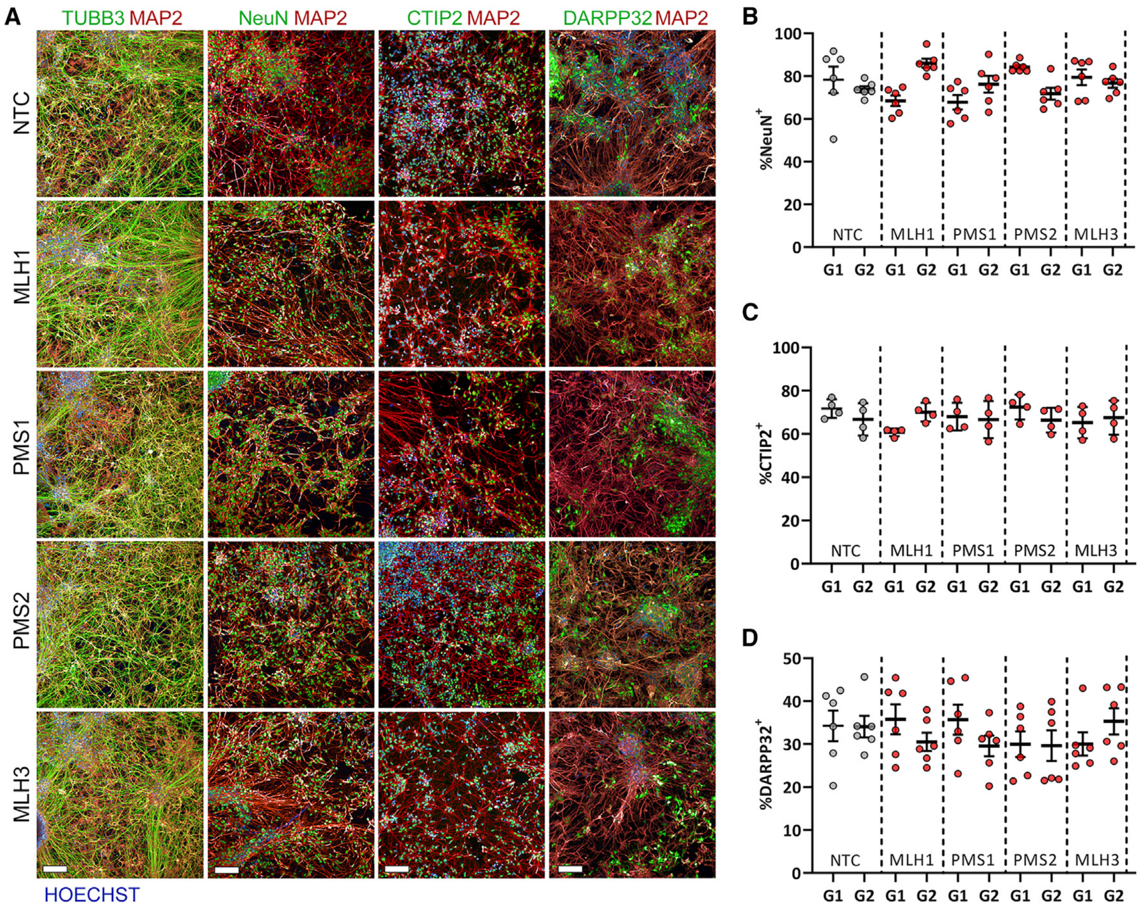

Figure 5. Constitutively lowering MMR factors does not affect iPSC competency to differentiate to a striatal identity
(A) Representative immunostaining for one of each indicated guides for the neuronal markers NeuN, TUBB3, and MAP2, as well as the striatal neuron marker CTIP2 and medium spiny neuron marker DARPP32. Scale bars, 100 μm.
(B–D) Quantification of marker positive cell frequency for NeuN (B), CTIP2 (C), and DARPP32 (D) as proportion of the total cells counterstained with Hoechst.
Five fields each from three replicate wells of two striatal differentiations with mean bar ±SEM for each targeting and non-targeting guide.

expression using an inducible strep-tagged variant of either MSH3 or MLH1 restored repeat expansion to wild-type rates (Figure S6).

Lowering of each MutL component slows somatic expansion in striatal neuron cultures

We next differentiated a subset of CRISPRi iPSCs to striatal neuron identity to investigate the effect on somatic expansion in populations of post-mitotic neurons. These differentiated cultures are mixed in nature with a predominant striatal identity and enriched for MSNs, a subtype known to be particularly vulnerable to repeat expansion.[4,64] From here on, these will be referred to as striatal neuron cultures for brevity. We did not pursue the effects of lowering MSH6 or LIG1 in neurons as neither showed a significant effect in iPSCs using this system. Although lowering MSH2 or MSH3 resulted in a robust slowing of repeat expansion in iPSCs, lowering of MSH2 *in vivo* is unlikely to be a viable long-term approach due its oncogenic potential.[65,66] In contrast, loss of MSH3 is substantially less oncogenic, particularly in the context of CNS cancers, and multiple approaches to therapeutic MSH3 lowering are currently under investigation.[44,45] We instead chose to focus on characterizing the effect of lowering the less-well-understood MutL factors—MLH1, PMS1, PMS2, and MLH3. Comparison of the expected frequency to actual occurrence of variants in MutL factors that are predicted to impair function in the population suggests variants in PMS1 and PMS2 to be well tolerated, as well as MLH3 and MLH1, but to a lesser degree (Figure S7).

Constitutive lowering of each MutL factor does not compromise the capacity of the iPSCs to differentiate into striatal neurons. Immunocytochemistry for key markers of neuronal and specifically striatal identity neurons showed robust and comparable frequencies between cultures of each set of lowered factors at day 100 (Figure 5A). Quantification of the post-mitotic neuron marker NeuN showed a consistent 70%–90% frequency between neuronal cultures of each genotype (Figure 5B). Similarly, quantification of the striatal neuron and MSN markers, CTIP2 and DARRP-32, ranged between 60%–80% and 30%–40% of the total population respectively

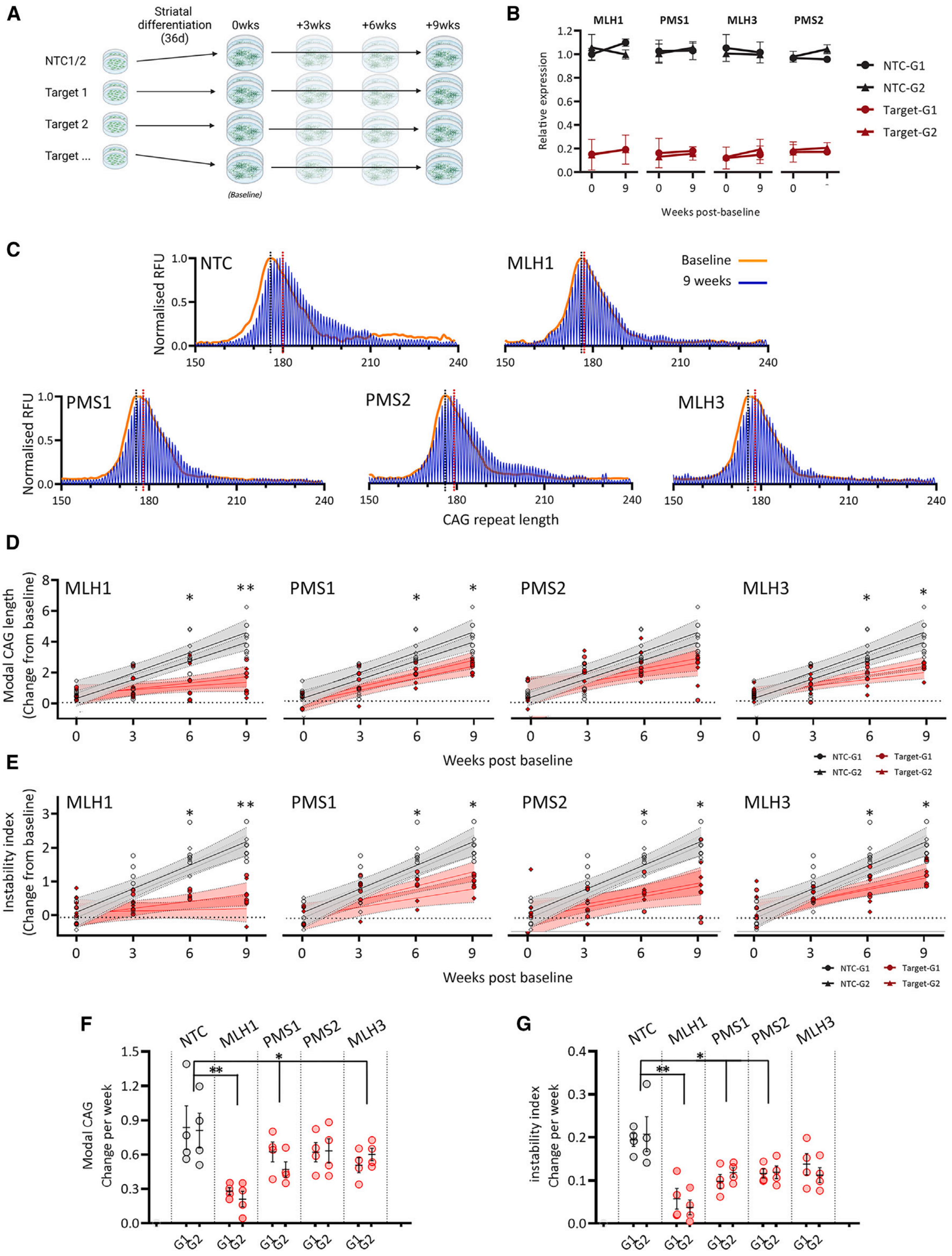

Figure 6. Reduced expression of each MutL factor slows somatic expansion in differentiated striatal neuron cultures

(A) Somatic instability was measured in cultures of striatal neurons differentiated from MLH1, PMS1, PMS2, MLH3, and non-targeting guide CRISPRi iPSC pools. CAG length baseline is set at 36 days post-neural induction.

(B) qPCR for each target shows lowering persists over the course. Mean expression relative to day zero ± SEM in triplicate for two NTC guides and the selected two target-specific guides at baseline and week 9.

(C) Representative fragment analysis traces for each MutL factor and NTC showing baseline (orange) and week 9 (blue). Dashed black vertical lines show modal baseline CAG size. Dashed red vertical lines show modal CAG size at endpoint.

(D) Modal CAG repeat lengths over a 9-week period for MLH1, PMS1, PMS2, and MLH3 lowered pools in comparison to NTC relative to baseline.

(legend continued on next page)

across each genotype (Figures 5C and 5D), as expected for the differentiation methods used.[46]

We used a time point 36 days post-initiation of differentiation as the baseline reference for assaying repeat expansion in striatal neuron cultures over a 9-week period (Figure 6A). Transcript levels were assayed at week 0 (baseline), and week 9 and showed that target lowering was maintained over the course of the experiment relative to the non-targeting controls (Figure 6B).

The change from the baseline modal repeat length and instability index relative to controls were quantified over this 9-week period. The modal CAG length in control striatal cultures carrying the NTC guides increased by an average of 4–5 additional repeats (Figures 6C and 6D) and showed an increase in overall instability by almost 2 units (Figure 6E). Lowering MutL in these striatal cultures led to a similar pattern of slowed repeat expansion as that seen in iPSCs. In cultures where MLH1 levels were lowered, around 1 repeat was added to the modal CAG length, while reduced levels of PMS1, PMS2, and MLH3 reduced expansion to 2–3 repeats over the same time course (Figures 6C and 6D). Comparison of the rate of modal CAG changes shows that reduced MLH1 expression slows repeat expansion by 69% ($p < 0.005$), while targeting PMS1, PMS2, and MLH3 slows it by 25%, 21%, and 28%, respectively ($p < 0.05$, Figure 6F).

These trends were maintained in the instability index (Figure 6G); while lowering MLH1 had the largest impact on reducing instability, an effect was also observed where PMS1, PMS2, and MLH3 levels were reduced. In NTC striatal cultures, instability index increased by an average of 0.20 units per week, and lowering MLH1 significantly reduced this by nearly 75% to around 0.05 units per week ($p < 0.005$, Figure 6G). Lowering expression of either PMS1, PMS2, or MLH3 also resulted in a lower instability but to a lesser degree with reductions of 46%, 41%, and 38%, respectively, compared to controls ($p < 0.05$, Figure 6G).

MMR factor levels are substantially reduced during differentiation to striatal neurons

While iPSCs maintain the necessarily robust DDR factor expression of human embryonic stem cell (hESC) and embryonic inner cell mass,[67] somatic cells typically express lower levels. This can be seen during the differentiation of the iPSCs to a striatal identity—loss of the pluripotency marker OCT4 and expression of both the post-mitotic neuronal marker NeuN and striatal marker CTIP2 is accompanied by substantial reductions in all MMR factor levels in differentiated cultures (Figure 7A).

PCNA has long been implicated in MMR in dividing cells,[68] and recent work has invoked PCNA as a requirement for CAG loop-out removal by FAN1 in competition with MutSβ using *in vitro* systems.[69] In contrast to dividing cells, PCNA is not typically observed in healthy post-mitotic neurons.[70] While PCNA was observable in bulk by western blot (Figure 7A), this signal likely comes predominantly from the small proportion of non-neuronal cells also present. Similar has been reported in bulk proteomics of HD iPSCs differentiated in a comparable manner.[71] We confirmed by immunostaining that very-low-to-undetectable levels of PCNA were present in mature CTIP2$^+$/DARPP-32$^+$ neurons at day 100. In contrast, substantially higher expression was seen at earlier stages in non-neuronal cells during neurogenesis (Figure 7B, D40).

Similarly, expression of MutS and LIG1 (Figure 7C) as well as MutL (Figure 7D) is significantly lower in striatal neurons (CTIP2/MAP2$^+$, white arrows) than non-neuronal cells (black arrows) in D100 cultures. LIG1 expression levels in striatal neurons appear higher, but levels remain less intense than those seen in non-neuronal levels in the same cultures (Figure 7C). Unlike the robust nuclear distribution seen for the other targets, MLH3 shows a distinct nucleocytoplasmic distribution, as previously reported in somatic and cancer cell lines.[72,73]

MMR co-factor protein levels are altered in addition to targeted factors

The levels of each protein in many multiprotein complexes such as MutS and MutL are regulated through the relative abundance of their component factors, with "orphan" excess co-factors targeted for degradation.[74] Therefore, to understand how the lowering of each target may influence repeat expansion, we must also look at the remaining levels of the relevant co-factors in the MutS and MutL complexes.[75–77] To characterize the extent to which altered co-factor abundance occurs because of targeted lowering, we quantified the protein levels of each MMR factor within each complex in iPSC cultures (Figures 7E and 7F). These data were collected from iPSC cultures due to the heterogeneous levels of expression observed in the striatal neuron cultures.

Targeting MSH2 expression reduced MSH2 protein levels to ~25% of control levels with either guide used (Figures 7E and 7G). This reduction in MSH2 was accompanied by a substantial decrease in MSH6 levels as well as a smaller decrease in MSH3 levels. In contrast, whereas MSH3 lowering reduced MSH3 protein levels down to 15%–29% of controls, the remaining MSH2 levels were not significantly reduced, and MSH6 levels remained unchanged. Targeted lowering of MSH6 resulted in a reduction of MSH6 protein to 31%–27% of control levels; a significant loss of MSH2 was seen compared to that

(E) Change in instability index over a 9-week period for MLH1, PMS1, PMS2, and MLH3 lowered pools in comparison to NTC relative to baseline.
Two differentiations of CRISPRi pools carrying one of two guides per target with four replicates. Dashed lines 95% confidence intervals.
(F and G) Rate of change in modal CAG per week (F) and change in instability index (G).
Open circles represent parallel cultures with mean bar ± SEM. *$p < 0.05$ **$p < 0.005$.

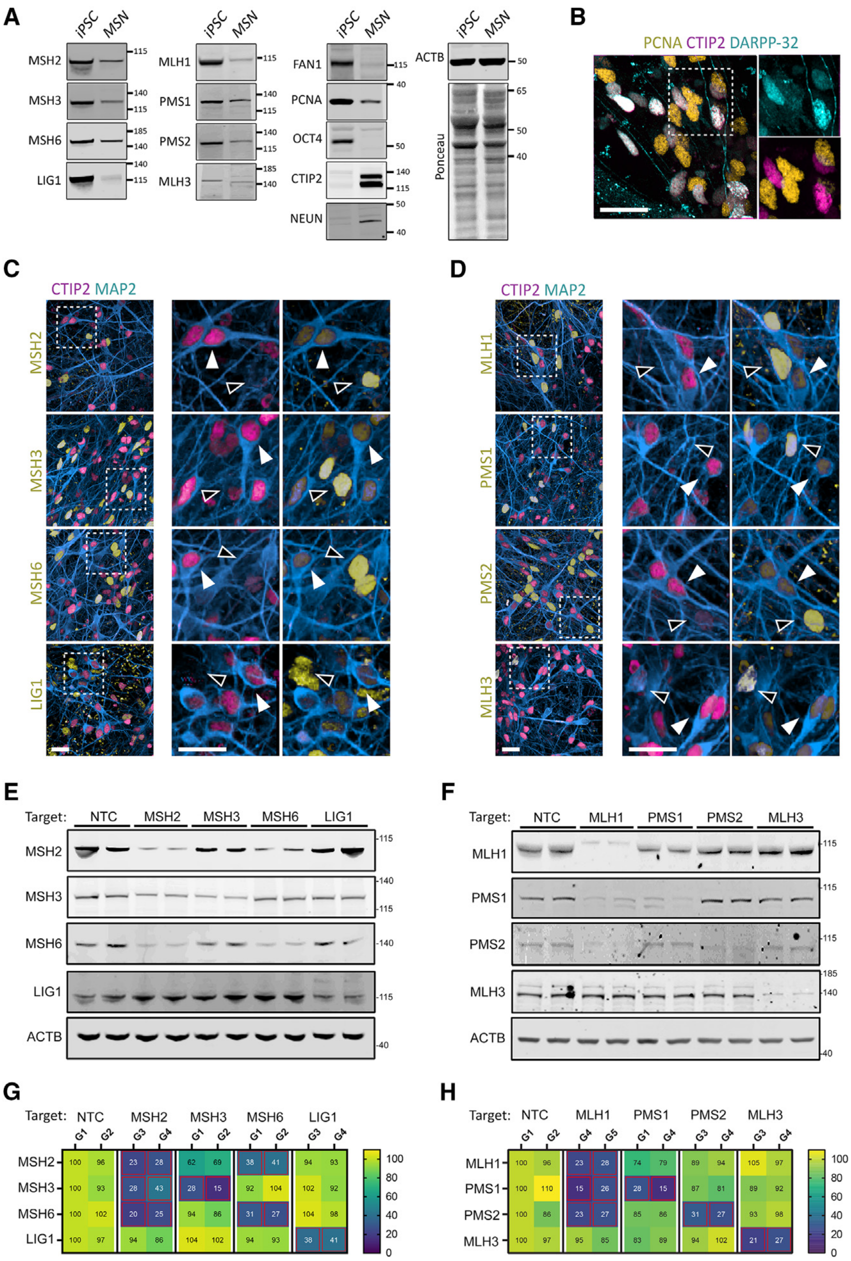

Figure 7. MMR factors are present at very low levels in medium spiny neurons and lowering expression impacts levels of partner proteins in iPSCs

(A) Western blot comparison protein levels in 125Q CRISPRi HD iPSC before (iPSC) and after striatal differentiation at day 100 (MSN).

(B) Immunostaining for PCNA, CTIP2, and DARPP-32 at intermediate stages of differentiation on day 40; dashed box indicates insets. Scale, 100 μm.

(C and D) Immunostaining for CTIP2 and MAP2 in conjunction with the indicated MMR factor on day 100; dashed box indicates inset location. Scale, 100 μm. White arrowheads: CTIP2/MAP2$^+$ MSNs; black arrowheads: MMR factor$^+$ non-neuronal cells.

(E and F) Western blot for MMR proteins in undifferentiated iPSCs from CRISPRi pools lowering expression of either MutS and LIG1 (E) or MutL (F) from two separate guides (pairs of columns).

(G and H) Heat maps for the corresponding blots quantifying the effects of lowering expression of the indicated target on protein level.

Mean of three blots, normalized to ACTB, and presented relative percentage of levels in the non-targeting control guide NTC-G1. Red boxes: $p < 0.05$.

associated with MSH3 lowering, down to 38%–41%. MSH6 lowering had no effect on MSH3 levels. The same pattern can be seen in U2OS knockout cell lines. The loss of MSH6 resulted in a significant reduction in the level of MSH2 protein but left MSH3 unaffected in U2OS (Figure S2A). Similarly, in MSH3 null U2OS, MSH2 levels showed a limited reduction.[22] Immunoprecipitation of MSH3 from MSH6 null U2OS showed that the remaining levels of MutSβ were unchanged (Figure S5D). LIG1 protein levels in iPSCs were reduced to around 40% where directly targeted, but this had no effect on MutS or MutL protein levels. Similarly, LIG1 levels remained unaffected by the lowering of any other MutS or MutL target (Figure 7G).

A similar pattern of reduction in co-factor levels occurs where components of MutL are targeted in the HD iPSCs. Lowering MLH1 reduced protein levels to 23%–28% between the 2 guides used relative to control guides (Figures 7F and 7H). This was accompanied by significant reductions in the levels of both PMS1 (15%–26% remaining) and PMS2 (23%–27% remaining), but no significant effect was seen on MLH3 levels (Figure 7H). Again, this was also observed in U2OS MLH1 null cells where PMS2 is almost undetectable and MLH3 levels remain unaffected (Figure S2A). Lowering either PMS1 or PMS2 in iPSCs also resulted in a reduction of MLH1 levels, with a larger effect on MLH1 levels seen where PMS1 was lowered (Figure 7H).

Discussion

Genetic studies to identify disease modifiers in human populations also offer valuable insight into the therapeutically exploitable tolerability of reduced gene function. Using disease-relevant cell types, we have shown that MMR-associated modifiers of HD onset and progression play a role in repeat expansion dynamics at the expanded *HTT* CAG repeat, as do MMR-associated factors not highlighted in GWASs. We also show that lowering rather than complete ablation of gene expression is sufficient to attenuate repeat expansion in these models.

MutSβ has been shown to be an almost essential driver of somatic expansion in multiple models across multiple repeat expansion-associated disorders including HD, and MSH3 locus modifiers are associated with delayed onset and progression.[20,44] We confirm this also occurs in our human *ex vivo* models where lowering either MSH2 or MSH3 has a dramatic effect on repeat expansion in our HD iPSCs.

In contrast, MSH6 (and so MutSα) has not been shown to affect somatic expansion in HD mouse models, though it has been implicated in preventing intergenerational contractions in CAG repeat length.[78] In a mouse models of myotonic dystrophy type 1 (DM1), loss of MSH6 led to increased expansion in non-neuronal tissue.[36] Here, we found no significant change in repeat expansion upon lowering MSH6. Although we were able to detect a significant MMR defect, we cannot discount the possibility that MSH6 levels simply weren't lowered sufficiently for a change in repeat expansion rate to become detectable. The same may be the case for LIG1, which also showed no significant effect upon lowering, and functional redundancy may occur for LIG1 through other ligases.[79,80] Data from *Lig1* mouse models suggest this redundancy is more effective where protein levels are reduced in comparison to deleterious LIG1 variants as *Lig1* null mice fare better than *Lig1* mutant mice.[81] This is consistent with an onset-delaying modifier haplotype in LIG1 resulting in a deleterious missense mutation while another is associated with increased expression.[82]

The missense MLH1 mutation I219V identified by GWASs is classed as benign, and no evidence for changes in transcript levels was found,[2] nor does it appear to affect protein stability.[75] Despite this, the effect we observe upon lowering MLH1 expression is concordant with the reduced repeat expansion reported in multiple Mlh1 null mouse models of HD[23,39] and our previous work in a U2OS cell system.[22] The same effect has also been seen on expansion of the GAA repeat in FRDA models.[42,83] One could ascribe this effect entirely to depletion of the total MutL pool in the absence of MLH1; however, we found that independently lowering each MutL co-factor, PMS1, PMS2, and MLH3, also slowed repeat expansion to a lesser degree despite their reported functional differences.

Individually, each of the MutL complexes has been shown to be required for expansion of the Fmr1 CGG-tract in a mouse embryonic stem cell (mESC) model of fragile X-related disorders (FXDs [MIM: 309550])[38]; however, the consistency of effect between different cell and repeat types is not well characterized. A role for PMS1 in repeat expansion has not been previously examined either at the HD CAG repeat tract or in human models. Onset hastening and delaying modifier haplotypes were identified, though their effect on PMS1 function are unclear.[2,82] Variants that are predicted to be deleterious to PMS1 function have been associated with a later AaO and reduced disease severity in HD cohorts through exome sequencing.[84] Here, we show lowering PMS1 levels slows CAG repeat expansion in human HD iPSC-derived striatal neurons.

In keeping with each MutL complex being required for repeat expansion, we found that lowering MLH3 expression was also sufficient to slow expansion despite MLH3 abundance (and so MutLγ) being substantially lower than both MutLα and MutLβ here and in other somatic cell types.[85] In FRDA and HD carrier fibroblasts, an HD mouse model and an FRDA model cell line, endonuclease-dead MLH3 variants result in the abrogation of the relevant repeat expansion to the same degree as a complete knockout.[42,86,87] Despite its clear role in repeat expansion, no modifier haplotypes are reported to associate with MLH3 in the Genetic Modifiers of Huntington's Disease (GeM-HD) Consortium GWASs.[2] MLH3 variants are, however, associated with somatic instability in blood in HD populations.[1] The lack of significance in the GWASs could be due to a low tolerance for reduced function MLH3 variants in germ cells during meiosis.[88,89]

We also show here that lowering PMS2 slows expansion of the *HTT* CAG repeat tract in both dividing iPSCs and mixed striatal neuron cultures. Again, modifier haplotypes have been associated both with early or delayed onset,[2] and previous characterizations of PMS2 have reported roles in both suppression and promotion of expansion. These observations have been made primarily for the mouse ortholog *Pms2* in the context of non-HD trinucleotide repeat tracts. Loss of *Pms2* slowed expansion of the CTG repeat in the brain of a DM1 mouse model and was accompanied by rare large contractions.[41] Here, we found that lowering PMS2 slowed expansion by around 21% in line with previous reports, which showed a 50% reduction in Pms2 null mice.[41] A more dramatic effect was seen on the *Fmr1* CGG repeat where loss of Pms2 completely halted expansion in FXD mESCs.[38] Conversely, no significant change was seen in the expansion rate of a construct carrying the FRDA GAA repeat in HEK cells where PMS2 levels were lowered by shRNA.[42] Bourn et al.[40] found that PMS2 supresses expansion in the brain of an FRDA mouse model—loss of PMS2 resulted in increased frequency of large expansions.

This apparent plasticity of PMS2's role in somatic instability could be due to cell type, repeat structure, or MutL abundance. One potential explanation is the competition model[90] where it's suggested that changes in repeat substrate length and MutLα/β/γ stoichiometry result in favoring either expansion, contraction, or maintenance. Within this model, the loss of PMS2 and so MutLα supresses expansion at increased repeat lengths, and the reduced levels here in our system may be sufficient to reach this theoretical threshold.

The implications of each MutL complex influencing repeat expansion rates are interesting mechanistically. PMS2 and MLH3, complexed with MLH1 as part of MutLα and MutLγ, are both independently capable of mediating repair in cell extracts otherwise lacking endogenous MutL.[30,91] While this appears to suggest redundancy between them, PMS1 is unable to mediate repair in MutL-deficient extracts.[92] Loss of either PMS2 or MLH3 in mouse

leads to high tumor burdens despite MLH3 being substantially less abundant than either PMS1 or PMS2.[85,93] Altering the relative levels of the different MutL complexes could affect the stoichiometry of MutS:MutL and MutLα:β:γ binding to DNA lesions.[94,95]

The role each of the different MMR complexes play at repeat structures is not well understood. Recent work characterized the effects of each on genome instability in a large panel of isogenic knockout hPSCs using whole-genome sequencing.[96] In this system, loss of MSH2, MSH6, MLH1, and PMS1, to a lesser degree, results in very similar insertion/deletion (indel) signatures with deletions frequently found at long repetitive sequences. In contrast, PMS2 favors both insertions and deletions at the same class of sequence. How this data correlates with effect at locus-specific expansions was not examined, but these repair signatures highlight the distinct effect of losing PMS2 in comparison to the other MMR factors.

Interpreting the loss of any individual MutS or MutL target is mechanistically difficult due to differential secondary effects on the levels of their heterodimer partners. Where MSH2 levels are reduced by CRISPRi, we observed a loss of a larger proportion of MSH6 than MSH3, as previously described in yeast and human cells.[76,97] While MSH3 has been reported to function outside of MMR in other DDR pathways, it is always as part of MutSβ.[98,99] This may suggest MSH3 has a higher affinity than MSH6 for MSH2, resulting in a bias toward MutSβ formation where MSH2 is lowered. Regardless, the net reduction in MutSβ here is still sufficient to attenuate expansion rates, as seen in heterozygous *Msh3* knockout or *Msh3* small interfering RNA (siRNA)-treated mice.[45,78] The reciprocal effect was seen upon lowering either MSH3 or MSH6; however, MSH2 levels were more substantially reduced where MSH6 levels were targeted, fitting with the higher abundance of MutSα than MutSβ.[100]

Similarly, where MLH1 levels are lowered, it is intuitive to assume proportional lowering of the function of MLH1-containing MutL complexes. This is supported by the MMR deficiency observed in response to MNNG. However, it is complicated by MLH3 stability being independent of MLH1 abundance (here, and in Cannavo et al.[85]), particularly in neurons as MutLγ (MLH1:MLH3) does not absolutely require PCNA for loading onto DNA for activity in cell-free systems.[30] This draws parallels to the stability of MSH3 upon lowering MSH2. However, unlike MSH3, MLH3 (and PMS2) has been reported to function independently of the MLH1 complexes.[101] These effects could be dissected by lowering multiple targets at once. As lowering each non-MLH1 MutL component slowed expansion to some degree, it also raises the question as to whether these effects are synergistic and whether we would see additive expansion rate slowing by targeting multiple components.

Single-cell transcriptomic and repeat sizing in postmortem striatal tissue from HD and SCA3 (MIM: 109150) donors have clearly shown MSNs are exquisitely vulnerable to CAG repeat expansion in both the mutant *HTT* and *ATXN3* (MIM: 607047) allele.[8] MSN-specific increases in MSH2 and MSH3 protein and transcript levels were also reported. Here, we found expression of MMR factors to be at lower levels in differentiated striatal neurons in comparison to levels in undifferentiated iPSCs and non-neuronal cells present within the differentiated cultures. The same effect has been described in hESC models of HD and DM1 upon differentiation to osteoprogenitor-like cells.[102] Reduced MMR gene expression was accompanied by reduced repeat instability in the differentiated cells compared to their parent hESC. One corollary of hPSC-derived models is the juvenile nature of neurons generated. Transcriptomics have shown iPSC-derived neurons to be comparable to mid-gestational identities *in vivo*.[103,104] The high metabolic rate of neurons and associated increase of genotoxic by-products leaves neurons particularly vulnerable to DNA damage.[105] DDR gene expression is increased in response to damage,[106,107] and this increases alongside the accumulation of DNA damage in aging brains.[108,109] Oxidative stress has been linked to repeat expansion in R6/1 mESCs with impaired double-stranded break (DSB) repair.[110] Perhaps transient increases in MMR factor expression in response to DNA damage act to briefly licence expansion, a window that widens with aging. Similarly, if PCNA is present in MSNs in our cultures, it is at very low levels; however, increased PCNA expression in neurons has been reported in response to DNA damage, age, and neurodegeneration.[111] We do not yet know what minimal levels of any of these factors are required to permit repeat expansion in post-mitotic neurons. As such, the low levels of PCNA or MMR proteins we observe in striatal neurons by immunofluorescence do not preclude their involvement in the repeat expansion process. However, the contrast between the lower-level expression in the neuronal majority and higher expression in the non-neuronal minority confounds dissection of this process, warranting further careful analysis using single-cell or highly enriching methods that are currently out of the scope of this manuscript.

Regardless of the mechanism, MSH3, PMS1, PMS2, and LIG1 are all tolerant of LoF over a predicted 2-fold range, and their variants are not readily removed from the population by selective pressures.[112,113] MSH2 and MLH1 are unlikely to be good therapeutic targets in the CNS as their loss is associated with significantly increased risk of CNS cancers, as is the loss of PMS2 to a lesser degree.[114,115] In addition to this, targeting the apparently flexible influence of PMS2 on repeat expansion could prove to be a double-edged sword without further investigation. We further highlight PMS1 as a potential therapeutic target for slowing repeat expansion. In this report, we have shown that lowering expression of MMR factors identified as HD-onset modifiers to levels regularly achieved by current therapeutic modalities was sufficient to slow the pathogenic expansion of the *HTT* CAG repeat tract. This further lends support to the search for an intervention that could potentially delay onset and progression in HD and other repeat expansion disorders that demonstrate somatic instability.

Data and code availability

This study did not generate any datasets. Code for repeat sizing by fragment analysis is available from the corresponding author on request.

Supplemental information

Supplemental information can be found online at https://doi.org/10.1016/j.ajhg.2024.04.015.

Acknowledgments

S.J.T. received funding from the CHDI Foundation. She is also partly supported by the UK Dementia Research Institute, which is funded by the UK Medical Research Council, Alzheimer's Society, and Alzheimer's Research UK. R.F. and R.G. were funded by the CHDI Foundation.

The U2OS cell line was kindly gifted by Prof. John Rouse (University of Dundee, Scotland).

We would like to thank the CHDI Foundation and UK Dementia Research Institute, which receives its funding from the UK Medical Research Council, Alzheimer's Society, and Alzheimer's Research UK. The authors thank all S.J.T. laboratory members, as well as Thomas Vogt, Brinda Prasad, Michael Finley, and other members of the CHDI foundation for helpful discussions.

Author contributions

S.J.T. supervised and led the work. S.J.T., M.F., and R.F. conceived the project. R.F. designed the experiments and performed iPSC and neuronal culture and associated assays and analyses. R.G. performed all U2OS cell culture and associated assays and analyses. M.F. and L.C. performed and analyzed fragment analysis. S.J.T. and M.F. secured funding. The manuscript was written by R.F., with editing by all co-authors.

Declaration of interests

In the past year, through the offices of UCL Consultants Ltd., a wholly owned subsidiary of University College London, S.J.T. has undertaken consultancy services for Alnylam Pharmaceuticals, Annexon, Ascidian Therapeutics, Arrowhead Pharmaceuticals, Atalanta Therapeutics, Design Therapeutics, F. Hoffman-La Roche, HCD Economics, IQVIA, Iris Medicine, Latus Bio, LifeEdit, Novartis Pharma, Pfizer, Prilenia Neurotherapeutics, PTC Therapeutics, Rgenta Therapeutics, Takeda Pharmaceuticals, UniQure Biopharma, and Vertex Pharmaceuticals. In the past 12 months, University College London Hospitals NHS Foundation Trust, S.J.T.'s host clinical institution, received funding to run clinical trials for F. Hoffman-La Roche, Novartis Pharma, PTC Therapeutics, and UniQure Biopharma.

Received: November 30, 2023
Accepted: April 18, 2024
Published: May 14, 2024

References

1. Ciosi, M., Maxwell, A., Cumming, S.A., Hensman Moss, D.J., Alshammari, A.M., Flower, M.D., Durr, A., Leavitt, B.R., Roos, R.A.C., et al.; TRACK-HD team (2019). A genetic association study of glutamine-encoding DNA sequence structures, somatic CAG expansion, and DNA repair gene variants, with Huntington disease clinical outcomes. EBioMedicine *48*, 568–580.
2. Genetic Modifiers of Huntington's Disease GeM-HD Consortium Electronic address gusella@helixmghharvardedu; and Genetic Modifiers of Huntington's Disease GeM-HD Consortium (2019). CAG Repeat Not Polyglutamine Length Determines Timing of Huntington's Disease Onset. Cell *178*, 887–900.e14.
3. The Huntington's Disease Collaborative Research Group (1993). A novel gene containing a trinucleotide repeat that is expanded and unstable on Huntington's disease chromosomes. Cell *72*, 971–983.
4. Swami, M., Hendricks, A.E., Gillis, T., Massood, T., Mysore, J., Myers, R.H., and Wheeler, V.C. (2009). Somatic expansion of the Huntington's disease CAG repeat in the brain is associated with an earlier age of disease onset. Hum. Mol. Genet. *18*, 3039–3047.
5. Wexler, N.S., Lorimer, J., Porter, J., Gomez, F., Moskowitz, C., Shackell, E., Marder, K., Penchaszadeh, G., Roberts, S.A., Gayán, J., et al. (2004). Venezuelan kindreds reveal that genetic and environmental factors modulate Huntington's disease age of onset. Proc. Natl. Acad. Sci. USA *101*, 3498–3503.
6. Donaldson, J., Powell, S., Rickards, N., Holmans, P., and Jones, L. (2021). What is the Pathogenic CAG Expansion Length in Huntington's Disease? J. Huntingtons Dis. *10*, 175–202.
7. Albin, R.L. (1995). Selective neurodegeneration in Huntington's disease. Ann. Neurol. *38*, 835–836.
8. Mätlik, K., Baffuto, M., Kus, L., Deshmukh, A.L., Davis, D.A., Paul, M.R., Carroll, T.S., Caron, M.C., Masson, J.Y., Pearson, C.E., and Heintz, N. (2024). Cell-type-specific CAG repeat expansions and toxicity of mutant Huntingtin in human striatum and cerebellum. Nat. Genet. *56*, 383–394.
9. Morigaki, R., and Goto, S. (2017). Striatal Vulnerability in Huntington's Disease: Neuroprotection Versus Neurotoxicity. Brain Sci. *7*, 63.
10. Pressl, C., Mätlik, K., Kus, L., Darnell, P., Luo, J.D., Paul, M.R., Weiss, A.R., Liguore, W., Carroll, T.S., Davis, D.A., et al. (2024). Selective vulnerability of layer 5a corticostriatal neurons in Huntington's disease. Neuron *112*, 924–941.e10.
11. Tabrizi, S.J., Langbehn, D.R., Leavitt, B.R., Roos, R.A., Durr, A., Craufurd, D., Kennard, C., Hicks, S.L., Fox, N.C., Scahill, R.I., et al. (2009). Biological and clinical manifestations of Huntington's disease in the longitudinal TRACK-HD study: cross-sectional analysis of baseline data. Lancet Neurol. *8*, 791–801.
12. Vonsattel, J.-P., Myers, R.H., Stevens, T.J., Ferrante, R.J., Bird, E.D., and Richardson, E.P., Jr. (1985). Neuropathological Classification of Huntington's Disease. J. Neuropathol. Exp. Neurol. *44*, 559–577.
13. Gonitel, R., Moffitt, H., Sathasivam, K., Woodman, B., Detloff, P.J., Faull, R.L.M., and Bates, G.P. (2008). DNA instability in postmitotic neurons. Proc. Natl. Acad. Sci. USA *105*, 3467–3472.
14. Kennedy, L., Evans, E., Chen, C.M., Craven, L., Detloff, P.J., Ennis, M., and Shelbourne, P.F. (2003). Dramatic tissue-specific mutation length increases are an early molecular event in Huntington disease pathogenesis. Hum. Mol. Genet. *12*, 3359–3367.

15. Kacher, R., Lejeune, F.X., Noël, S., Cazeneuve, C., Brice, A., Humbert, S., and Durr, A. (2021). Propensity for somatic expansion increases over the course of life in Huntington disease. Elife *10*, e64674.
16. Shelbourne, P.F., Keller-McGandy, C., Bi, W.L., Yoon, S.R., Dubeau, L., Veitch, N.J., Vonsattel, J.P., Wexler, N.S., US-Venezuela Collaborative Research Group, Arnheim, N., and Augood, S.J. (2007). Triplet repeat mutation length gains correlate with cell-type specific vulnerability in Huntington disease brain. Hum. Mol. Genet. *16*, 1133–1142.
17. Wright, G.E.B., Collins, J.A., Kay, C., McDonald, C., Dolzhenko, E., Xia, Q., Bečanović, K., Drögemöller, B.I., Semaka, A., Nguyen, C.M., et al. (2019). Length of Uninterrupted CAG, Independent of Polyglutamine Size, Results in Increased Somatic Instability, Hastening Onset of Huntington Disease. Am. J. Hum. Genet. *104*, 1116–1126.
18. Bettencourt, C., Hensman-Moss, D., Flower, M., Wiethoff, S., Brice, A., Goizet, C., Stevanin, G., Koutsis, G., Karadima, G., Panas, M., et al. (2016). DNA repair pathways underlie a common genetic mechanism modulating onset in polyglutamine diseases. Ann. Neurol. *79*, 983–990.
19. Genetic Modifiers of Huntington's Disease GeM-HD Consortium (2015). Identification of Genetic Factors that Modify Clinical Onset of Huntington's Disease. Cell *162*, 516–526.
20. Moss, D.J.H., Pardiñas, A.F., Langbehn, D., Lo, K., Leavitt, B.R., Roos, R., Durr, A., Mead, S., et al.; TRACK-HD investigators; and REGISTRY investigators (2017). Identification of genetic variants associated with Huntington's disease progression: a genome-wide association study. Lancet Neurol. *16*, 701–711.
21. Goold, R., Flower, M., Moss, D.H., Medway, C., Wood-Kaczmar, A., Andre, R., Farshim, P., Bates, G.P., Holmans, P., Jones, L., and Tabrizi, S.J. (2019). FAN1 modifies Huntington's disease progression by stabilizing the expanded HTT CAG repeat. Hum. Mol. Genet. *28*, 650–661.
22. Goold, R., Hamilton, J., Menneteau, T., Flower, M., Bunting, E.L., Aldous, S.G., Porro, A., Vicente, J.R., Allen, N.D., Wilkinson, H., et al. (2021). FAN1 controls mismatch repair complex assembly via MLH1 retention to stabilize CAG repeat expansion in Huntington's disease. Cell Rep. *36*, 109649.
23. Loupe, J.M., Pinto, R.M., Kim, K.H., Gillis, T., Mysore, J.S., Andrew, M.A., Kovalenko, M., Murtha, R., Seong, I., Gusella, J.F., et al. (2020). Promotion of somatic CAG repeat expansion by Fan1 knock-out in Huntington's disease knock-in mice is blocked by Mlh1 knock-out. Hum. Mol. Genet. *29*, 3044–3053.
24. Manley, K., Shirley, T.L., Flaherty, L., and Messer, A. (1999). Msh2 deficiency prevents in vivo somatic instability of the CAG repeat in Huntington disease transgenic mice. Nat. Genet. *23*, 471–473.
25. Genschel, J., Littman, S.J., Drummond, J.T., and Modrich, P. (1998). Isolation of MutSbeta from human cells and comparison of the mismatch repair specificities of MutSbeta and MutSalpha. J. Biol. Chem. *273*, 19895–19901.
26. Iyer, R.R., Pluciennik, A., Burdett, V., and Modrich, P.L. (2006). DNA mismatch repair: functions and mechanisms. Chem. Rev. *106*, 302–323.
27. Lang, W.H., Coats, J.E., Majka, J., Hura, G.L., Lin, Y., Rasnik, I., and McMurray, C.T. (2011). Conformational trapping of Mismatch Recognition Complex MSH2/MSH3 on repair-resistant DNA loops. Proc. Natl. Acad. Sci. USA *108*, E837–E844.
28. Iyer, R.R., and Pluciennik, A. (2021). DNA Mismatch Repair and its Role in Huntington's Disease. J. Huntingtons Dis. *10*, 75–94.
29. Genschel, J., Kadyrova, L.Y., Iyer, R.R., Dahal, B.K., Kadyrov, F.A., and Modrich, P. (2017). Interaction of proliferating cell nuclear antigen with PMS2 is required for MutLα activation and function in mismatch repair. Proc. Natl. Acad. Sci. USA *114*, 4930–4935.
30. Kadyrova, L.Y., Gujar, V., Burdett, V., Modrich, P.L., and Kadyrov, F.A. (2020). Human MutLγ, the MLH1–MLH3 heterodimer, is an endonuclease that promotes DNA expansion. Proc. Natl. Acad. Sci. USA *117*, 3535–3542.
31. Keogh, N., Chan, K.Y., Li, G.-M., and Lahue, R.S. (2017). MutSβ abundance and Msh3 ATP hydrolysis activity are important drivers of CTG•CAG repeat expansions. Nucleic Acids Res. *45*, 10068–10078.
32. Lee, J.-M., Zhang, J., Su, A.I., Walker, J.R., Wiltshire, T., Kang, K., Dragileva, E., Gillis, T., Lopez, E.T., Boily, M.J., et al. (2010). A novel approach to investigate tissue-specific trinucleotide repeat instability. BMC Syst. Biol. *4*, 29.
33. Owen, B.A.L., Yang, Z., Lai, M., Gajec, M., Badger, J.D., 2nd, Hayes, J.J., Edelmann, W., Kucherlapati, R., Wilson, T.M., and McMurray, C.T. (2005). (CAG)(n)-hairpin DNA binds to Msh2-Msh3 and changes properties of mismatch recognition. Nat. Struct. Mol. Biol. *12*, 663–670.
34. Tomé, S., Holt, I., Edelmann, W., Morris, G.E., Munnich, A., Pearson, C.E., and Gourdon, G. (2009). MSH2 ATPase Domain Mutation Affects CTG•CAG Repeat Instability in Transgenic Mice. PLoS Genet. *5*, e1000482.
35. Wheeler, V.C., Lebel, L.A., Vrbanac, V., Teed, A., te Riele, H., and MacDonald, M.E. (2003). Mismatch repair gene Msh2 modifies the timing of early disease in Hdh(Q111) striatum. Hum. Mol. Genet. *12*, 273–281.
36. van den Broek, W.J.A.A., Nelen, M.R., Wansink, D.G., Coerwinkel, M.M., te Riele, H., Groenen, P.J.T.A., and Wieringa, B. (2002). Somatic expansion behaviour of the (CTG)n repeat in myotonic dystrophy knock-in mice is differentially affected by Msh3 and Msh6 mismatch-repair proteins. Hum. Mol. Genet. *11*, 191–198.
37. Du, J., Campau, E., Soragni, E., Ku, S., Puckett, J.W., Dervan, P.B., and Gottesfeld, J.M. (2012). Role of mismatch repair enzymes in GAA·TTC triplet-repeat expansion in Friedreich ataxia induced pluripotent stem cells. J. Biol. Chem. *287*, 29861–29872.
38. Miller, C.J., Kim, G.-Y., Zhao, X., and Usdin, K. (2020). All three mammalian MutL complexes are required for repeat expansion in a mouse cell model of the Fragile X-related disorders. PLoS Genet. *16*, e1008902.
39. Pinto, R.M., Dragileva, E., Kirby, A., Lloret, A., Lopez, E., St Claire, J., Panigrahi, G.B., Hou, C., Holloway, K., Gillis, T., et al. (2013). Mismatch Repair Genes Mlh1 and Mlh3 Modify CAG Instability in Huntington's Disease Mice: Genome-Wide and Candidate Approaches. PLoS Genet. *9*, e1003930.
40. Bourn, R.L., De Biase, I., Pinto, R.M., Sandi, C., Al-Mahdawi, S., Pook, M.A., and Bidichandani, S.I. (2012). Pms2 Suppresses Large Expansions of the (GAA·TTC)n Sequence in Neuronal Tissues. PLoS One *7*, e47085.
41. Gomes-Pereira, M., Fortune, M.T., Ingram, L., McAbney, J.P., and Monckton, D.G. (2004). Pms2 is a genetic enhancer of trinucleotide CAG.CTG repeat somatic mosaicism: implications for the mechanism of triplet repeat expansion. Hum. Mol. Genet. *13*, 1815–1825.

42. Halabi, A., Fuselier, K.T.B., and Grabczyk, E. (2018). GAA•TTC repeat expansion in human cells is mediated by mismatch repair complex MutLγ and depends upon the endonuclease domain in MLH3 isoform one. Nucleic Acids Res. *46*, 4022–4032.
43. Hong, E.P., MacDonald, M.E., Wheeler, V.C., Jones, L., Holmans, P., Orth, M., Monckton, D.G., Long, J.D., Kwak, S., Gusella, J.F., and Lee, J.M. (2021). Huntington's Disease Pathogenesis: Two Sequential Components. J. Huntingtons Dis. *10*, 35–51.
44. Flower, M., Lomeikaite, V., Ciosi, M., Cumming, S., Morales, F., Lo, K., Hensman Moss, D., Jones, L., Holmans, P., et al.; TRACK-HD Investigators (2019). MSH3 modifies somatic instability and disease severity in Huntington's and myotonic dystrophy type 1. Brain *142*, 1876–1886.
45. O'Reilly, D., Belgrad, J., Ferguson, C., Summers, A., Sapp, E., McHugh, C., Mathews, E., Boudi, A., Buchwald, J., Ly, S., et al. (2023). Di-valent siRNA-mediated silencing of MSH3 blocks somatic repeat expansion in mouse models of Huntington's disease. Mol. Ther. *31*, 1661–1674.
46. Arber, C., Precious, S.V., Cambray, S., Risner-Janiczek, J.R., Kelly, C., Noakes, Z., Fjodorova, M., Heuer, A., Ungless, M.A., Rodríguez, T.A., et al. (2015). Activin A directs striatal projection neuron differentiation of human pluripotent stem cells. Dev. Camb. Engl. *142*, 1375–1386.
47. Sanson, K.R., Hanna, R.E., Hegde, M., Donovan, K.F., Strand, C., Sullender, M.E., Vaimberg, E.W., Goodale, A., Root, D.E., Piccioni, F., and Doench, J.G. (2018). Optimized libraries for CRISPR-Cas9 genetic screens with multiple modalities. Nat. Commun. *9*, 5416.
48. Jiang, W., Hua, R., Wei, M., Li, C., Qiu, Z., Yang, X., and Zhang, C. (2015). An optimized method for high-titer lentivirus preparations without ultracentrifugation. Sci. Rep. *5*, 13875.
49. Tian, R., Gachechiladze, M.A., Ludwig, C.H., Laurie, M.T., Hong, J.Y., Nathaniel, D., Prabhu, A.V., Fernandopulle, M.S., Patel, R., Abshari, M., et al. (2019). CRISPR Interference-Based Platform for Multimodal Genetic Screens in Human iPSC-Derived Neurons. Neuron *104*, 239–255.e12.
50. Alerasool, N., Segal, D., Lee, H., and Taipale, M. (2020). An efficient KRAB domain for CRISPRi applications in human cells. Nat. Methods *17*, 1093–1096.
51. Ho, S.-M., Hartley, B.J., Flaherty, E., Rajarajan, P., Abdelaal, R., Obiorah, I., Barretto, N., Muhammad, H., Phatnani, H.P., Akbarian, S., and Brennand, K.J. (2017). Evaluating Synthetic Activation and Repression of Neuropsychiatric-Related Genes in hiPSC-Derived NPCs, Neurons, and Astrocytes. Stem Cell Rep. *9*, 615–628.
52. Cerbini, T., Funahashi, R., Luo, Y., Liu, C., Park, K., Rao, M., Malik, N., and Zou, J. (2015). Transcription activator-like effector nuclease (TALEN)-mediated CLYBL targeting enables enhanced transgene expression and one-step generation of dual reporter human induced pluripotent stem cell (iPSC) and neural stem cell (NSC) lines. PLoS One *10*, e0116032.
53. Li, X.-L., Li, G.H., Fu, J., Fu, Y.W., Zhang, L., Chen, W., Arakaki, C., Zhang, J.P., Wen, W., Zhao, M., et al. (2018). Highly efficient genome editing via CRISPR–Cas9 in human pluripotent stem cells is achieved by transient BCL-XL overexpression. Nucleic Acids Res. *46*, 10195–10215.
54. Gupta, D., Lin, B., Cowan, A., and Heinen, C.D. (2018). ATR-Chk1 activation mitigates replication stress caused by mismatch repair-dependent processing of DNA damage. Proc. Natl. Acad. Sci. USA *115*, 1523–1528.
55. Schindelin, J., Arganda-Carreras, I., Frise, E., Kaynig, V., Longair, M., Pietzsch, T., Preibisch, S., Rueden, C., Saalfeld, S., Schmid, B., et al. (2012). Fiji: an open-source platform for biological-image analysis. Nat. Methods *9*, 676–682.
56. Teo, C.R.L., Wang, W., Yang Law, H., Lee, C.G., and Chong, S.S. (2008). Single-step scalable-throughput molecular screening for Huntington disease. Clin. Chem. *54*, 964–972.
57. Mangiarini, L., Sathasivam, K., Seller, M., Cozens, B., Harper, A., Hetherington, C., Lawton, M., Trottier, Y., Lehrach, H., Davies, S.W., and Bates, G.P. (1996). Exon 1 of the HD gene with an expanded CAG repeat is sufficient to cause a progressive neurological phenotype in transgenic mice. Cell *87*, 493–506.
58. Gudmundsson, S., Singer-Berk, M., Watts, N.A., Phu, W., Goodrich, J.K., Solomonson, M., Genome Aggregation Database Consortium, Rehm, H.L., MacArthur, D.G., and O'Donnell-Luria, A. (2022). Variant interpretation using population databases: Lessons from gnomAD. Hum. Mutat. *43*, 1012–1030.
59. Schroering, A.G., Edelbrock, M.A., Richards, T.J., and Williams, K.J. (2007). The cell cycle and DNA mismatch repair. Exp. Cell Res. *313*, 292–304.
60. Li, G.-M. (2008). Mechanisms and functions of DNA mismatch repair. Cell Res. *18*, 85–98.
61. Duckett, D.R., Drummond, J.T., Murchie, A.I., Reardon, J.T., Sancar, A., Lilley, D.M., and Modrich, P. (1996). Human MutSalpha recognizes damaged DNA base pairs containing O6-methylguanine, O4-methylthymine, or the cisplatin-d(GpG) adduct. Proc. Natl. Acad. Sci. USA *93*, 6443–6447.
62. Cejka, P., Stojic, L., Mojas, N., Russell, A.M., Heinimann, K., Cannavó, E., di Pietro, M., Marra, G., and Jiricny, J. (2003). Methylation-induced G(2)/M arrest requires a full complement of the mismatch repair protein hMLH1. EMBO J. *22*, 2245–2254.
63. Yan, T., Berry, S.E., Desai, A.B., and Kinsella, T.J. (2003). DNA Mismatch Repair (MMR) Mediates 6-Thioguanine Genotoxicity by Introducing Single-strand Breaks to Signal a G2-M Arrest in MMR-proficient RKO Cells1. Clin. Cancer Res. *9*, 2327–2334.
64. Telenius, H., Kremer, B., Goldberg, Y.P., Theilmann, J., Andrew, S.E., Zeisler, J., Adam, S., Greenberg, C., Ives, E.J., and Clarke, L.A. (1994). Somatic and gonadal mosaicism of the Huntington disease gene CAG repeat in brain and sperm. Nat. Genet. *6*, 409–414.
65. Dominguez-Valentin, M., Sampson, J.R., Seppälä, T.T., Ten Broeke, S.W., Plazzer, J.P., Nakken, S., Engel, C., Aretz, S., Jenkins, M.A., Sunde, L., et al. (2020). Cancer risks by gene, age, and gender in 6350 carriers of pathogenic mismatch repair variants: findings from the Prospective Lynch Syndrome Database. Genet. Med. *22*, 15–25.
66. Therkildsen, C., Ladelund, S., Rambech, E., Persson, A., Petersen, A., and Nilbert, M. (2015). Glioblastomas, astrocytomas and oligodendrogliomas linked to Lynch syndrome. Eur. J. Neurol. *22*, 717–724.
67. Lin, B., Gupta, D., and Heinen, C.D. (2014). Human Pluripotent Stem Cells Have a Novel Mismatch Repair-dependent Damage Response. J. Biol. Chem. *289*, 24314–24324.
68. Umar, A., Buermeyer, A.B., Simon, J.A., Thomas, D.C., Clark, A.B., Liskay, R.M., and Kunkel, T.A. (1996). Requirement for PCNA in DNA Mismatch Repair at a Step Preceding DNA Resynthesis. Cell *87*, 65–73.
69. Phadte, A.S., Bhatia, M., Ebert, H., Abdullah, H., Elrazaq, E.A., Komolov, K.E., and Pluciennik, A. (2023). FAN1 removes

triplet repeat extrusions via a PCNA- and RFC-dependent mechanism. Proc. Natl. Acad. Sci. USA *120*, e2302103120.

70. Ino, H., and Chiba, T. (2000). Expression of proliferating cell nuclear antigen (PCNA) in the adult and developing mouse nervous system. Brain Res. Mol. Brain Res. *78*, 163–174.
71. Tshilenge, K.-T., Aguirre, C.G., Bons, J., Gerencser, A.A., Basisty, N., Song, S., Rose, J., Lopez-Ramirez, A., Naphade, S., Loureiro, A., et al. (2023). Proteomic Analysis of Huntington's Disease Medium Spiny Neurons Identifies Alterations in Lipid Droplets. Mol. Cell. Proteomics *22*, 100534.
72. Korhonen, M.K., Raevaara, T.E., Lohi, H., and Nyström, M. (2007). Conditional nuclear localization of hMLH3 suggests a minor activity in mismatch repair and supports its role as a low-risk gene in HNPCC. Oncol. Rep. *17*, 351–354.
73. Ou, J., Rasmussen, M., Westers, H., Andersen, S.D., Jager, P.O., Kooi, K.A., Niessen, R.C., Eggen, B.J.L., Nielsen, F.C., Kleibeuker, J.H., et al. (2009). Biochemical characterization of MLH3 missense mutations does not reveal an apparent role of MLH3 in Lynch syndrome. Genes Chromosomes Cancer *48*, 340–350.
74. Juszkiewicz, S., and Hegde, R.S. (2018). Quality Control of Orphaned Proteins. Mol. Cell *71*, 443–457.
75. Abildgaard, A.B., Stein, A., Nielsen, S.V., Schultz-Knudsen, K., Papaleo, E., Shrikhande, A., Hoffmann, E.R., Bernstein, I., Gerdes, A.M., Takahashi, M., et al. (2019). Computational and cellular studies reveal structural destabilization and degradation of MLH1 variants in Lynch syndrome. Elife *8*, e49138.
76. Arlow, T., Kim, J., Haye-Bertolozzi, J.E., Martínez, C.B., Fay, C., Zorensky, E., Rose, M.D., and Gammie, A.E. (2021). MutSα mismatch repair protein stability is governed by subunit interaction, acetylation, and ubiquitination. *G3 (Bethesda*. Md.) *11* (3).
77. Cannavo, E., Gerrits, B., Marra, G., Schlapbach, R., and Jiricny, J. (2007). Characterization of the Interactome of the Human MutL Homologues MLH1, PMS1, and PMS2. J. Biol. Chem. *282*, 2976–2986.
78. Dragileva, E., Hendricks, A., Teed, A., Gillis, T., Lopez, E.T., Friedberg, E.C., Kucherlapati, R., Edelmann, W., Lunetta, K.L., MacDonald, M.E., and Wheeler, V.C. (2009). Intergenerational and striatal CAG repeat instability in Huntington's disease knock-in mice involve different DNA repair genes. Neurobiol. Dis. *33*, 37–47.
79. Gazy, I., Hayward, B., Potapova, S., Zhao, X., and Usdin, K. (2019). Double-strand break repair plays a role in repeat instability in a fragile X mouse model. DNA Repair *74*, 63–69.
80. Masani, S., Han, L., Meek, K., and Yu, K. (2016). Redundant function of DNA ligase 1 and 3 in alternative end-joining during immunoglobulin class switch recombination. Proc. Natl. Acad. Sci. USA *113*, 1261–1266.
81. Bentley, D.J., Harrison, C., Ketchen, A.M., Redhead, N.J., Samuel, K., Waterfall, M., Ansell, J.D., and Melton, D.W. (2002). DNA ligase I null mouse cells show normal DNA repair activity but altered DNA replication and reduced genome stability. J. Cell Sci. *115*, 1551–1561.
82. Lee, J.-M., Huang, Y., Orth, M., Gillis, T., Siciliano, J., Hong, E., Mysore, J.S., Lucente, D., Wheeler, V.C., Seong, I.S., et al. (2022). Genetic modifiers of Huntington disease differentially influence motor and cognitive domains. Am. J. Hum. Genet. *109*, 885–899.
83. Ezzatizadeh, V., Sandi, C., Sandi, M., Anjomani-Virmouni, S., Al-Mahdawi, S., and Pook, M.A. (2014). MutLα heterodimers modify the molecular phenotype of Friedreich ataxia. PLoS One *9*, e100523.
84. McAllister, B., Donaldson, J., Binda, C.S., Powell, S., Chughtai, U., Edwards, G., Stone, J., Lobanov, S., Elliston, L., Schuhmacher, L.N., et al. (2022). Exome sequencing of individuals with Huntington's disease implicates FAN1 nuclease activity in slowing CAG expansion and disease onset. Nat. Neurosci. *25*, 446–457.
85. Cannavo, E., Marra, G., Sabates-Bellver, J., Menigatti, M., Lipkin, S.M., Fischer, F., Cejka, P., and Jiricny, J. (2005). Expression of the MutL Homologue hMLH3 in Human Cells and its Role in DNA Mismatch Repair. Cancer Res. *65*, 10759–10766.
86. Hayward, B.E., Steinbach, P.J., and Usdin, K. (2020). A point mutation in the nuclease domain of MLH3 eliminates repeat expansions in a mouse stem cell model of the Fragile X-related disorders. Nucleic Acids Res. *48*, 7856–7863.
87. Roy, J.C.L., Vitalo, A., Andrew, M.A., Mota-Silva, E., Kovalenko, M., Burch, Z., Nhu, A.M., Cohen, P.E., Grabczyk, E., Wheeler, V.C., and Mouro Pinto, R. (2021). Somatic CAG expansion in Huntington's disease is dependent on the MLH3 endonuclease domain, which can be excluded via splice redirection. Nucleic Acids Res. *49*, 3907–3918.
88. Charbonneau, N., Amunugama, R., Schmutte, C., Yoder, K., and Fishel, R. (2009). Evidence that hMLH3 functions primarily in meiosis and in hMSH2-hMSH3 mismatch repair. Cancer Biol. Ther. *8*, 1411–1420.
89. Dai, J., Sanchez, A., Adam, C., Ranjha, L., Reginato, G., Chervy, P., Tellier-Lebegue, C., Andreani, J., Guérois, R., Ropars, V., et al. (2021). Molecular basis of the dual role of the Mlh1-Mlh3 endonuclease in MMR and in meiotic crossover formation. Proc. Natl. Acad. Sci. USA *118*, e2022704118.
90. Zhao, X., Kumari, D., Miller, C.J., Kim, G.Y., Hayward, B., Vitalo, A.G., Pinto, R.M., and Usdin, K. (2021). Modifiers of Somatic Repeat Instability in Mouse Models of Friedreich Ataxia and the Fragile X-Related Disorders: Implications for the Mechanism of Somatic Expansion in Huntington's Disease. J. Huntingtons Dis. *10*, 149–163.
91. Pluciennik, A., Burdett, V., Baitinger, C., Iyer, R.R., Shi, K., and Modrich, P. (2013). Extrahelical (CAG)/(CTG) triplet repeat elements support proliferating cell nuclear antigen loading and MutLα endonuclease activation. Proc. Natl. Acad. Sci. USA *110*, 12277–12282.
92. Räschle, M., Marra, G., Nyström-Lahti, M., Schär, P., and Jiricny, J. (1999). Identification of hMutLβ, a Heterodimer of hMLH1 and hPMS1. J. Biol. Chem. *274*, 32368–32375.
93. Chen, P.-C., Dudley, S., Hagen, W., Dizon, D., Paxton, L., Reichow, D., Yoon, S.R., Yang, K., Arnheim, N., Liskay, R.M., and Lipkin, S.M. (2005). Contributions by MutL Homologues Mlh3 and Pms2 to DNA Mismatch Repair and Tumor Suppression in the Mouse. Cancer Res. *65*, 8662–8670.
94. Bradford, K.C., Wilkins, H., Hao, P., Li, Z.M., Wang, B., Burke, D., Wu, D., Smith, A.E., Spaller, L., Du, C., et al. (2020). Dynamic human MutSα-MutLα complexes compact mismatched DNA. Proc. Natl. Acad. Sci. USA *117*, 16302–16312.
95. Elez, M., Radman, M., and Matic, I. (2012). Stoichiometry of MutS and MutL at unrepaired mismatches in vivo suggests a mechanism of repair. Nucleic Acids Res. *40*, 3929–3938.
96. Zou, X., Koh, G.C.C., Nanda, A.S., Degasperi, A., Urgo, K., Roumeliotis, T.I., Agu, C.A., Badja, C., Momen, S., Young, J., et al. (2021). A systematic CRISPR screen defines mutational mechanisms underpinning signatures caused by

replication errors and endogenous DNA damage. Nat. Can. (Ott.) *2*, 643–657.

97. Sakellariou, D., Bak, S.T., Isik, E., Barroso, S.I., Porro, A., Aguilera, A., Bartek, J., Janscak, P., and Peña-Diaz, J. (2022). MutSβ regulates G4-associated telomeric R-loops to maintain telomere integrity in ALT cancer cells. Cell Rep. *39*, 110602.
98. Eichmiller, R., Medina-Rivera, M., DeSanto, R., Minca, E., Kim, C., Holland, C., Seol, J.H., Schmit, M., Oramus, D., Smith, J., et al. (2018). Coordination of Rad1–Rad10 interactions with Msh2–Msh3, Saw1 and RPA is essential for functional 3′ non-homologous tail removal. Nucleic Acids Res. *46*, 5075–5096.
99. Oh, J.-M., Kang, Y., Park, J., Sung, Y., Kim, D., Seo, Y., Lee, E.A., Ra, J.S., Amarsanaa, E., Park, Y.U., et al. (2023). MSH2-MSH3 promotes DNA end resection during homologous recombination and blocks polymerase theta-mediated end-joining through interaction with SMARCAD1 and EXO1. Nucleic Acids Res. *51*, 5584–5602.
100. Reyes, G.X., Schmidt, T.T., Kolodner, R.D., and Hombauer, H. (2015). New insights into the mechanism of DNA mismatch repair. Chromosoma *124*, 443–462.
101. Pannafino, G., and Alani, E. (2021). Coordinated and Independent Roles for MLH Subunits in DNA Repair. Cells *10*, 948.
102. Seriola, A., Spits, C., Simard, J.P., Hilven, P., Haentjens, P., Pearson, C.E., and Sermon, K. (2011). Huntington's and myotonic dystrophy hESCs: down-regulated trinucleotide repeat instability and mismatch repair machinery expression upon differentiation. Hum. Mol. Genet. *20*, 176–185.
103. Handel, A.E., Chintawar, S., Lalic, T., Whiteley, E., Vowles, J., Giustacchini, A., Argoud, K., Sopp, P., Nakanishi, M., Bowden, R., et al. (2016). Assessing similarity to primary tissue and cortical layer identity in induced pluripotent stem cell-derived cortical neurons through single-cell transcriptomics. Hum. Mol. Genet. *25*, 989–1000.
104. Mehta, S.R., Tom, C.M., Wang, Y., Bresee, C., Rushton, D., Mathkar, P.P., Tang, J., and Mattis, V.B. (2018). Human Huntington's Disease iPSC-Derived Cortical Neurons Display Altered Transcriptomics, Morphology, and Maturation. Cell Rep. *25*, 1081–1096.e6.
105. Hegde, M.L., Mantha, A.K., Hazra, T.K., Bhakat, K.K., Mitra, S., and Szczesny, B. (2012). Oxidative genome damage and its repair: implications in aging and neurodegenerative diseases. Mech. Ageing Dev. *133*, 157–168.
106. Belloni, M., Uberti, D., Rizzini, C., Ferrari-Toninelli, G., Rizzonelli, P., Jiricny, J., Spano, P., and Memo, M. (1999). Distribution and kainate-mediated induction of the DNA mismatch repair protein MSH2 in rat brain. Neuroscience *94*, 1323–1331.
107. Tennen, R.I., Haye, J.E., Wijayatilake, H.D., Arlow, T., Ponzio, D., and Gammie, A.E. (2013). Cell-cycle and DNA damage regulation of the DNA mismatch repair protein Msh2 occurs at the transcriptional and post-transcriptional level. DNA Repair *12*, 97–109.
108. Lombard, D.B., Chua, K.F., Mostoslavsky, R., Franco, S., Gostissa, M., and Alt, F.W. (2005). DNA Repair, Genome Stability, and Aging. Cell *120*, 497–512.
109. Lu, T., Pan, Y., Kao, S.Y., Li, C., Kohane, I., Chan, J., and Yankner, B.A. (2004). Gene regulation and DNA damage in the ageing human brain. Nature *429*, 883–891.
110. Jonson, I., Ougland, R., Klungland, A., and Larsen, E. (2013). Oxidative stress causes DNA triplet expansion in Huntington's disease mouse embryonic stem cells. Stem Cell Res. *11*, 1264–1271.
111. Frade, J.M., and Ovejero-Benito, M.C. (2015). Neuronal cell cycle: the neuron itself and its circumstances. Cell Cycle *14*, 712–720.
112. Karczewski, K.J., Weisburd, B., Thomas, B., Solomonson, M., Ruderfer, D.M., Kavanagh, D., Hamamsy, T., Lek, M., Samocha, K.E., Cummings, B.B., et al. (2017). The ExAC browser: displaying reference data information from over 60 000 exomes. Nucleic Acids Res. *45*, D840–D845.
113. Kosmicki, J.A., Samocha, K.E., Howrigan, D.P., Sanders, S.J., Slowikowski, K., Lek, M., Karczewski, K.J., Cutler, D.J., Devlin, B., Roeder, K., et al. (2017). Refining the role of de novo protein-truncating variants in neurodevelopmental disorders by using population reference samples. Nat. Genet. *49*, 504–510.
114. Caccese, M., Ius, T., Simonelli, M., Fassan, M., Cesselli, D., Dipasquale, A., Cavallin, F., Padovan, M., Salvalaggio, A., Gardiman, M.P., et al. (2020). Mismatch-Repair Protein Expression in High-Grade Gliomas: A Large Retrospective Multicenter Study. Int. J. Mol. Sci. *21*, 6716.
115. Sijmons, R.H., and Hofstra, R.M.W. (2016). Review: Clinical aspects of hereditary DNA Mismatch repair gene mutations. DNA Repair *38*, 155–162.

The American Journal of Human Genetics, Volume *111*

Supplemental information

Therapeutic validation of MMR-associated genetic modifiers in a human *ex vivo* model of Huntington disease

Ross Ferguson, Robert Goold, Lucy Coupland, Michael Flower, and Sarah J. Tabrizi

SUPPLEMENTAL MATERIALS

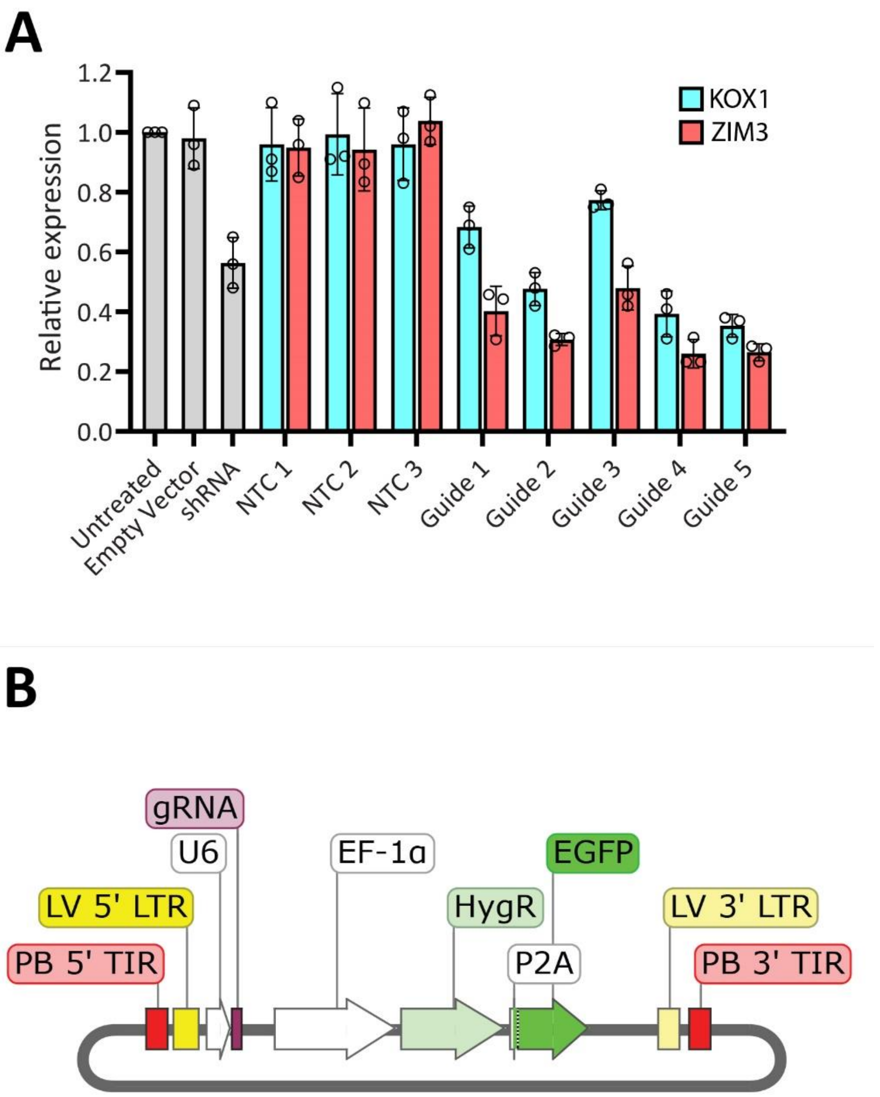

Figure S1 – A more effective repressor construct and guide delivery vector. Comparison between KOX1 and ZIM3 KRAB domains indicated better or same knock-down using PMS2 targeting guides (A). Guide sequences were cloned into either pLentiguide GFP-HYG or a modified version with additional PiggyBAC terminal inverted repeats (B).

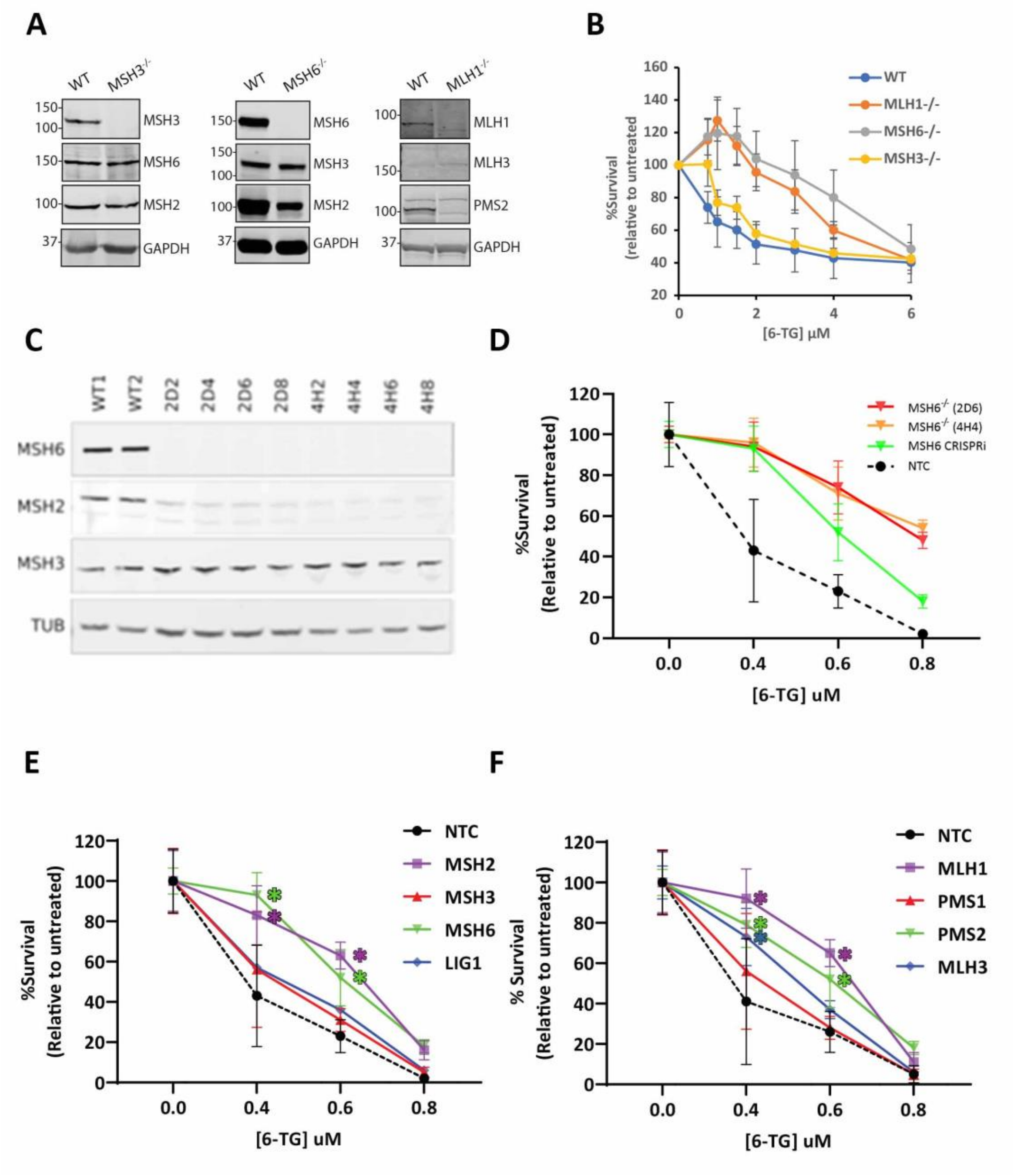

Figure S2 – Knockout of MMR factors in U2OS cells results in MMR deficiency.

Western blot for MMR associated proteins in U2OS MSH3, MSH6 & MLH1 knockout clones (-/-), compared to wild type U2OS (WT) (A). MMR deficiency seen after 6-thioguanine treatment of MSH3, MSH6 and MLH1 null and wildtype U2OS cells (B). Western blot of MSH6 knockout 125Q iPSCs generated using the same strategy with deletions in exon 2 or 4 (C). MSH6 knockout iPSC (clones 2D6 & 4H4) show further reduced sensitivity to 6-TG treatment than CRISPRi lowered MSH6 iPSCs (D). iPSCs show high vulnerability to 6-TG with a small window for seeing functional differences between genotype in MutS (E), or MutL (F) CRISPRi iPSCs. *P<0.05.

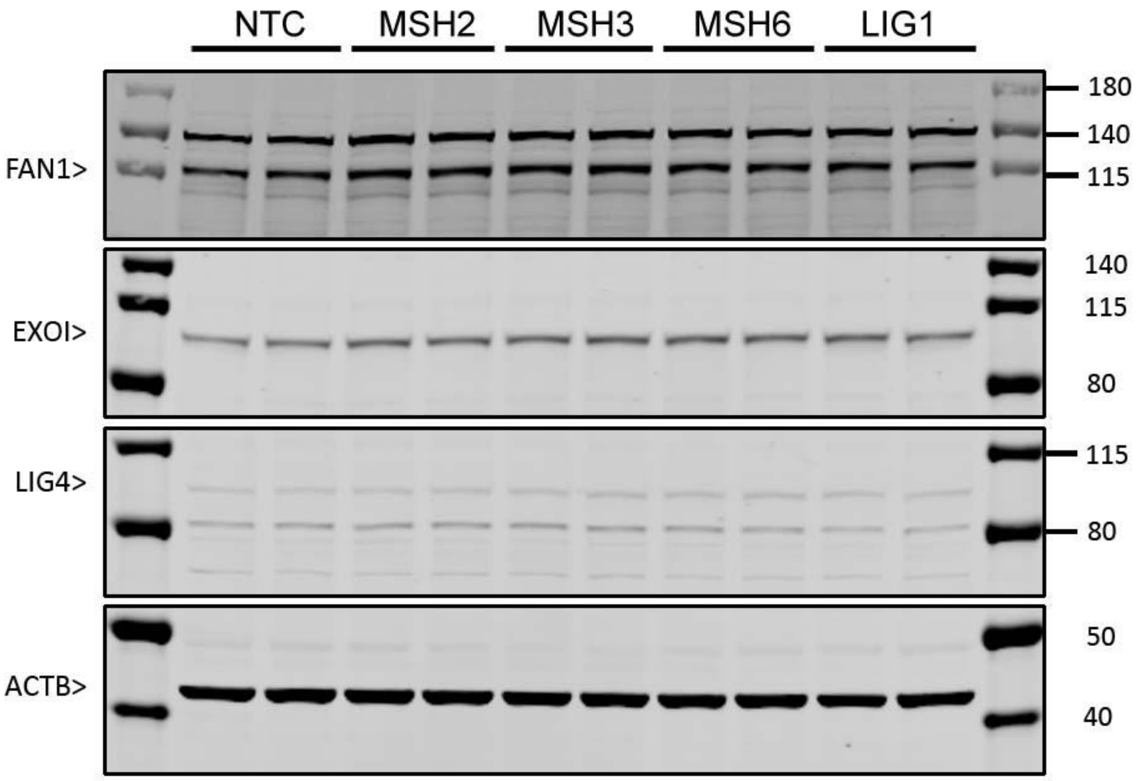

Figure S3 – No changes seen in LIG4, EXO1 or FAN1 in CRISPRi MutS pools. Western blots for indicated proteins (left) in two different CRISPRi pools targeting the indicated MutS factors and LIG1.

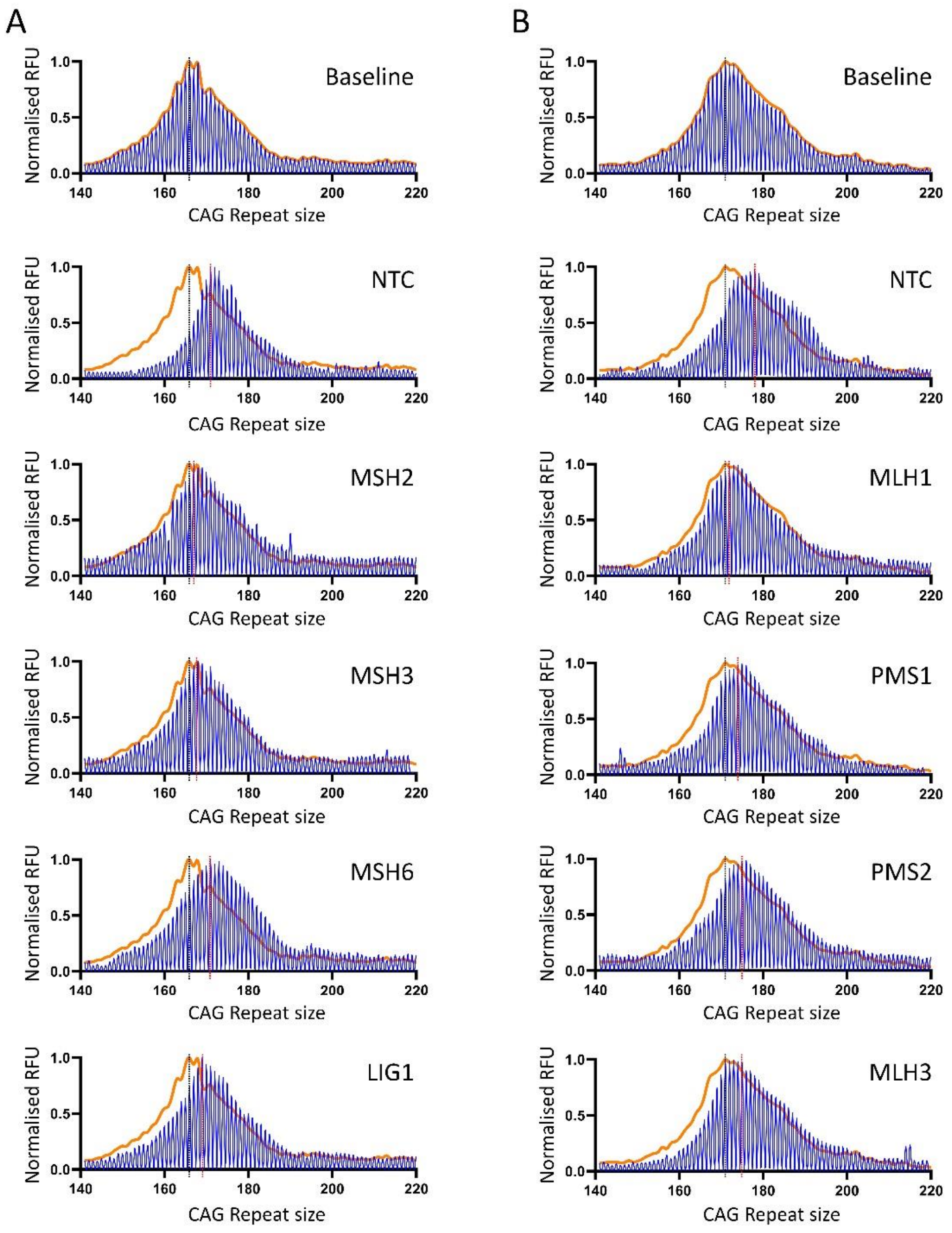

Figure S4 – Representative fragment analysis traces for CRISPRi iPSCs.

Representative traces with peak height normalised to the modal peak for baseline samples (D0) or endpoint (D80) for each of the CRISPRi pools where MutS & LIG1 (A), or MutL (B) are lowered. Dashed black vertical lines show modal baseline CAG size. Dashed red vertical lines show modal CAG size at endpoint. Orange trace shows the baseline from each pool for reference in each panel

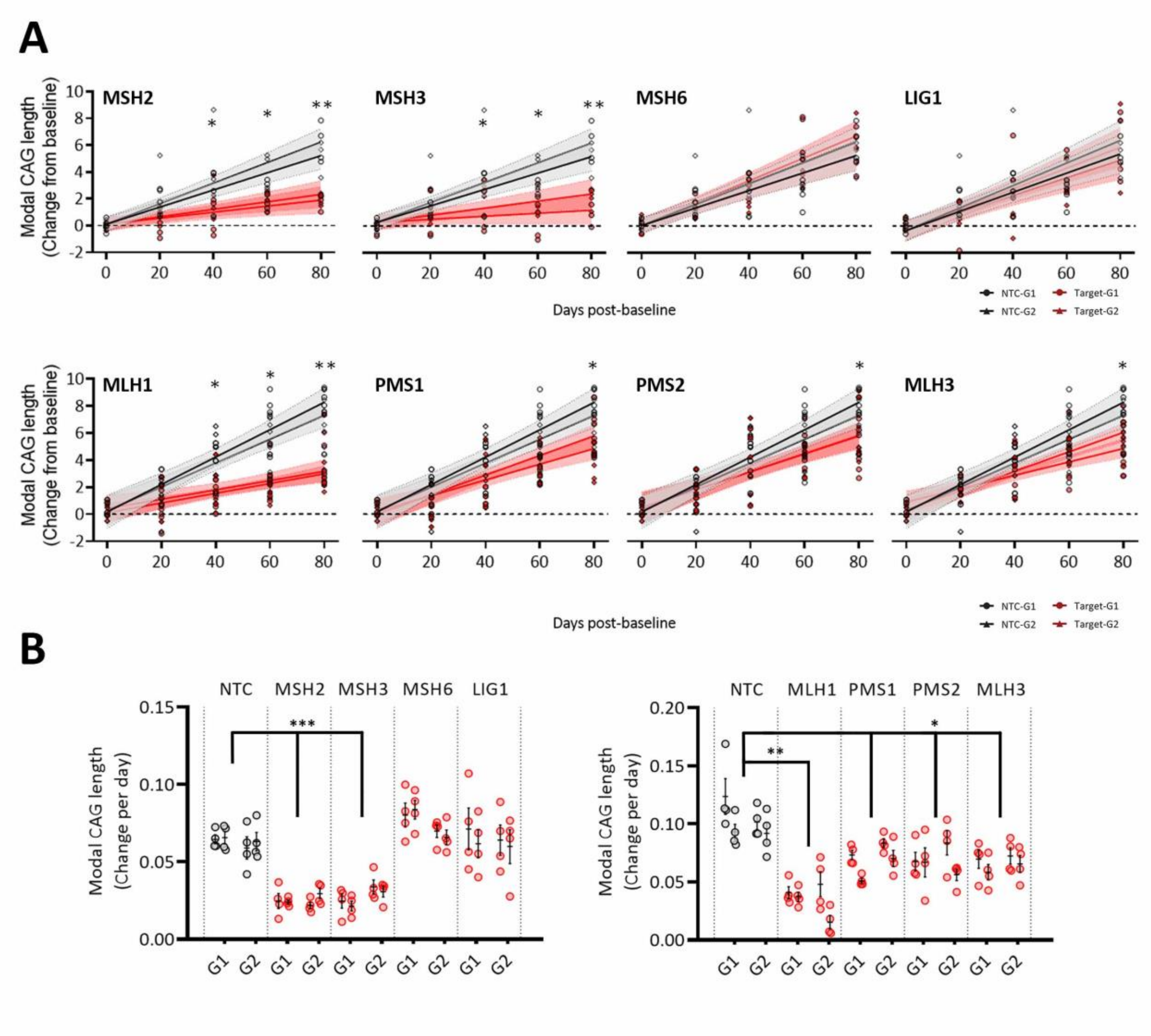

Figure S5 - Reduced expression of MutS and MutL components slows increase in modal repeat length in dividing iPSCs. Change in modal CAG repeat length over an eighty-day period for MutS & LIG1 (A), and MutL (B) lowered cultures relative to baseline on D0. Four cultures per guide passaged in parallel each from two independent CRISPRi pools, dashed lines 95% confidence intervals. Rate of change in modal repeat length per day for MutS & LIG1 (C), and MutL (D). Open circles represent parallel cultures with mean bar ±SEM. * P<0.05 * P<0.005.

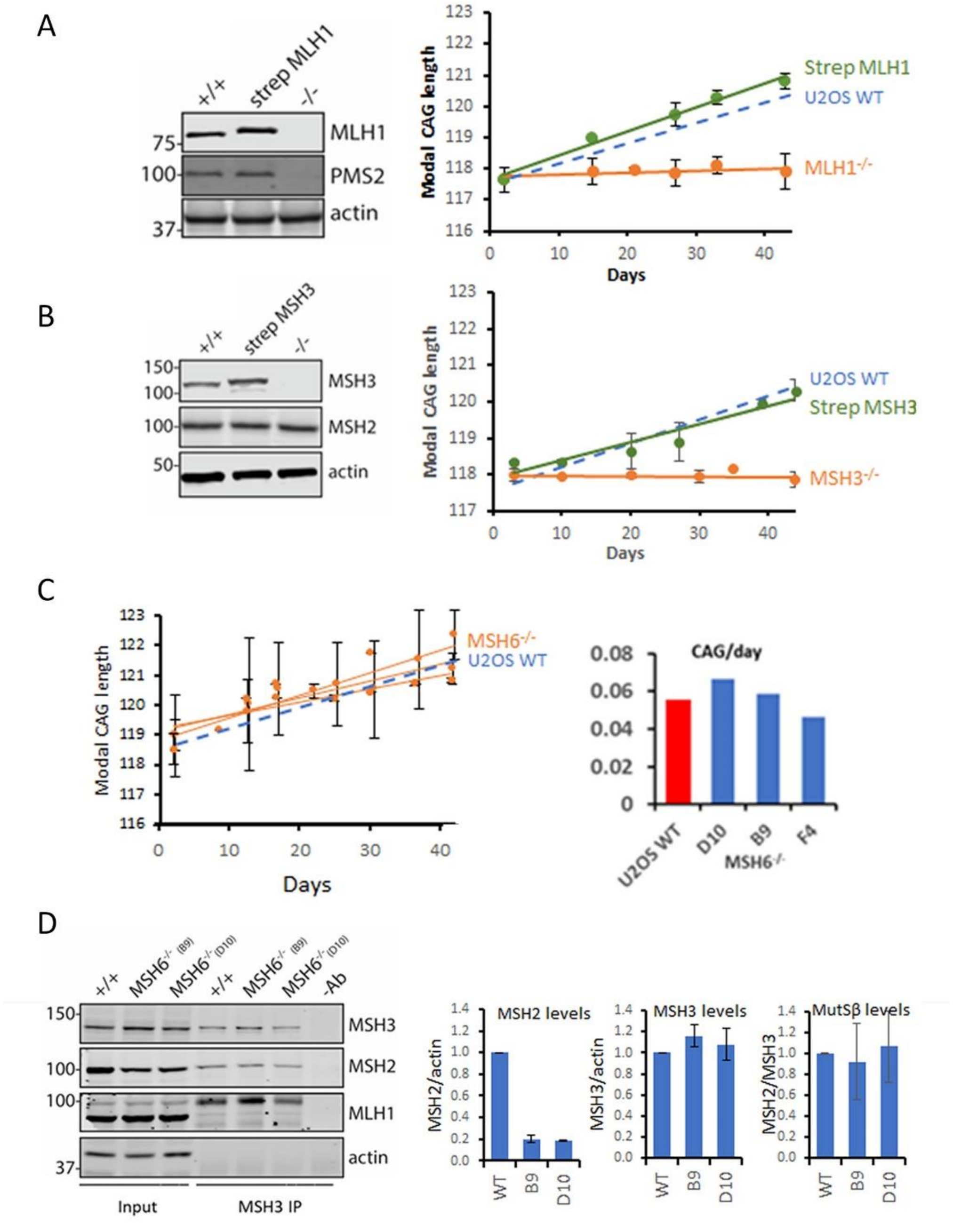

Figure S6 – Repeat expansion in MSH3, MLH1 and MSH6 null U2OS. A 118Q repeat construct introduced by transduction shows an increase in modal length over 40 days. Expansion is halted in MLH1$^{-/-}$ U2OS cells but can be rescued to WT U2OS cells rates by adding back a strep-tagged MLH1 to near physiological levels by dox-inducible expression (A). Expansion is halted and rescued in MSH3$^{-/-}$ U2OS cells using the same system (B). No change repeat expansion is observed in MSH6$^{-/-}$ U2OS cells (C). Immunoprecipitation shows that while MSH2 levels are lowered in MSH6$^{-/-}$ cells there is no change in MSH3/MSH2 interactions (D)

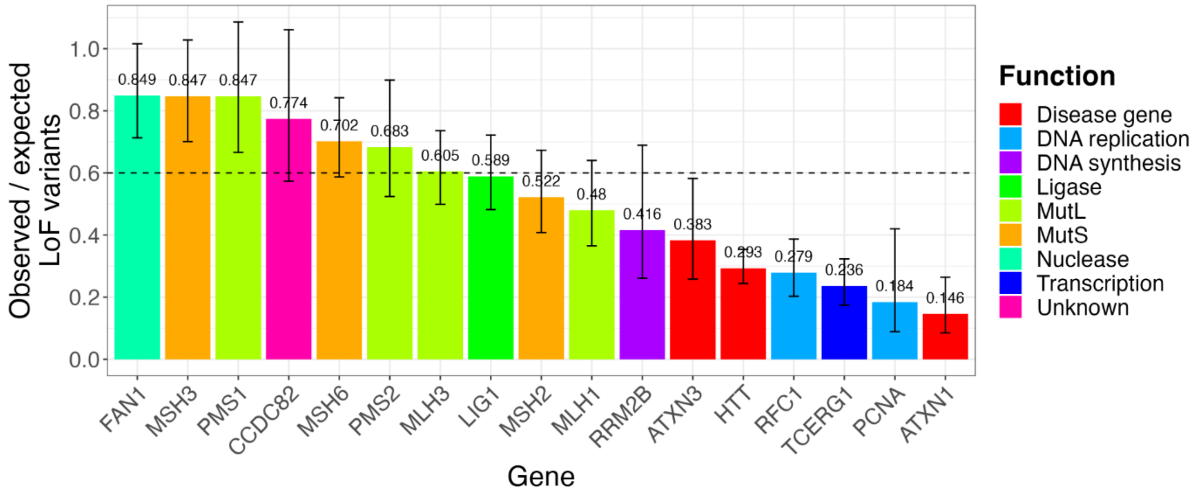

Figure S7 – Genetic constraint in selected HD modifier and DNA repair genes. Bar chart of loss-of-function metrics for selected genes, based on data from gnomAD (Gudmundsson et al., 2022). The y-axis represents the ratio of observed / expected ratio of loss-of-function variants (pLoF) for each gene, using GENCODE v39 annotations and GRCh38 reference. Error bars represent the 90% confidence interval. Lower o/e values indicate genes are under stronger selection. An o/e ratio of 0.2, for example, means there were 20% of the expected number of variants observed in that gene. Bars falling below the horizontal dashed line at an o/e ratio of 0.6 are generally considered to be under a high degree of genetic constraint, with significant selection against loss-of-function variation.

Table S1 – CRISPRi guide sequences

Target	Guide	Sequence (5'-3')
**NTC**	1	TCTCTCGGAGTGGAGCAACA
**NTC**	2	GTCAGGTAATAGTCGGACTC
**NTC**	3	CGCAATCCCTTAGGATAGCC
**NTC**	4	ATCTACAATCCAGCCCTCTA
**MSH2**	1	GAGGTGAGGAGGTTTCGACA
**MSH2**	2	GTGAGGAGGTTTCGACATGG
**MSH2**	3	CGACATGGCGGTGCAGCCGA
**MSH2**	4	GACGCTGCAGTTGGAGAGCG
**MSH3**	1	AGGATGGCAGCCCGGCGGCA
**MSH3**	2	GGGCAAGGATGGCAGCCCGG
**MSH3**	3	GAGACATGGCAGGGCAAGGA
**MSH3**	4	GCTTCCGGCGAGACATGGCA
**MSH6**	1	AAAGCACCGCATCTACCGCG
**MSH6**	2	CAGGAGCCGCGCGGTAGATG
**MSH6**	3	GGCCCAACCGTTCTGTCGGA
**MSH6**	4	GCTCCGTCCGACAGAACGGT
**LIG1**	1	GCAGTCCCAAGTTCGCGCCA
**LIG1**	2	GAATGCCGTGGCGCGAACTT
**LIG1**	3	GCGCGAACTTGGGACTGCAG
**LIG1**	4	GCAACACACTCAGATCCGCC
**MLH1**	1	TCGGCGGCTGGACGAGACAG
**MLH1**	2	CCAAAATGTCGTTCGTGGCA
**MLH1**	3	CAAAATGTCGTTCGTGGCAG
**MLH1**	4	TCGTGGCAGGGGTTATTCGG
**MLH1**	5	TGGCGCCAAAATGTCGTTCG
**PMS1**	1	ACTACCTTCCTGCTAGCGCG
**PMS1**	2	GCGCTAGCAGGAAGGTAGTG
**PMS1**	3	ATTGCCTGCCTCGCGCTAGC
**PMS1**	4	GGCTGCTTGCGGCTAGTGGA
**PMS1**	5	CGCTGGCTGCTTGCGGCTAG
**PMS2**	1	CGGATCGGGTGTTGCATCCA
**PMS2**	2	AGCTGAGAGCTCGAGGTGAG
**PMS2**	3	CGAGCTCTCAGCTCGCTCCA
**PMS2**	4	ACGAGATCGCTGCAACACTG
**PMS2**	5	AACACTGAGGTCGCCACTCC
**MLH3**	1	AAGATCCAAGGTGCGCGCGT
**MLH3**	2	GTGTCGGAGAATTTGTTAAG
**MLH3**	3	TGTCGGAGAATTTGTTAAGC
**MLH3**	4	TTCTCCGACACCAACCGCCT
**MLH3**	5	TTCTCCGACACCAACCGCCT

Table S2 – Primer sequences

Primer	Sequence (5'-3')	Amplicon
**pLG_cPCR_F1**	TTTCAGACCCACCTCCCAAC	2447bp +Stuffer
**pLG_cPCR_R1**	AGTGGATCTCTGCTGTCCCT	558bp - Stuffer
**pLG_cPCR_F2**	ATCGTTTCAGACCCACCTCC	2242bp +Stuffer
**pLG_cPCR_R2**	ATCGTTTCAGACCCACCTCC	353bp - Stuffer
**PB 5P TIR - pLG**	cttcccaacagttgcCATGCGTCAATTTTACGCATGATTATCTTTAACGTACGTCACAATATGATTATCTTTCTAGGGttaaagcctgaatggcgaatgg	1751bp
**PB 3P TIR - pLG**	ttcgccagttaatagtttgcCATGCGTCAATTTTACGCAGACTATCTTTCTAGGGTTAAgtctgacgctcagtggaacg	
**ZIM3_5P_HindIII**	cacaagcttaattctggctaactgtcggg	398bp
**ZIM3_3P_Stop_MluI**	aattcacgcgtctaaaccactttgtacaagaaagttgggtagag	
**ZNF80_F1**	TGCAGCTCATCCTCACTTGG	436bp
**ZNF80_R1**	GAGGCAAGGCCTTTGTACCT	
**GPR15_F1**	CTTGCATGAGTGTTGACCGC	303bp
**GPR15_R1**	AATGGGCACACAGCTTCCTT	
**C13_5PJ_F1**	TATCGGTATCCTCGACTTGGC	1843bp
**C13_5PJ_R1**	CTCCTCCACGTCACCGCA	
**C13_3PJ_F1**	GCAACCTGTTCAGTGCGTC	1975bp
**C13_3PJ_R1**	AGTATGCTTATGCCAAGCCA	
**dCas9_F1**	CATCGAGCAGATCAGCGAGT	275bp
**dCas9_R1**	CGATCCGTGTCTCGTACAGG	
**T7_MSH6_Ex2_F1**	GCCTTTTTCCTGCCATCAGC	647bp
**T7_MSH6_Ex2_R1**	TTTCACAACTGCCACCCCTT	
**T7_MSH6_Ex4_F1**	TTGGCATATGAAGTTGCAGCATA	642bp
**T7_MSH6_Ex4_R1**	GCTGTTCAGGCCTTCACTCT	

Table S3 – Knockout guides

MSH6 (NG_007111.1)	Sequence (5'-3')	Cut position
**Hs.Cas9.MSH6.1.AF**	AGCCUAAGACACAAGGAUCUGUUUUAGAGCUAUGCU	20548
**(Targeting)**	AGCCTAAGACACAAGGATCTAGG	
**Hs.Cas9.MSH6.1.AH**	AUUUAAGCCAGACACUAAGGGUUUUAGAGCUAUGCU	20645
**(Targeting)**	ATTTAAGCCAGACACTAAGGAGG	

Table S4 – TaqMan Assays

Target	Assay ID
**ATP5B**	Hs00969569_m1
**EIF4A2**	Hs00756996_g1
**UBC**	Hs00824723_m1
**MLH1**	Hs00179866_m1
**PMS1**	Hs00922262_m1
**PMS2**	Hs00241053_m1
**MLH3**	Hs00998142_m1
**MSH2**	Hs00953527_m1
**MSH3**	Hs00989003_m1
**MSH6**	Hs00943000_m1
**LIG1**	Hs01553527_m1

Table S5 – Antibodies

Primary	Supplier	Cat#	Dilution
**MLH1**	BD	#554073	1:500 (ICC), 1:1000 (WB)
**PMS1**	Invitrogen	PA5-86724	1:500 (ICC), 1:1000 (WB)
**PMS2**	Invitrogen	PA5-87127	1:500 (ICC), 1:1000 (WB)
**MLH3**	Santa Cruz	sc-25313	1:500 (ICC), 1:1000 (WB)
**MLH3**	ProteinTech	25298-1-AP	1:500 (ICC), 1:1000 (WB)
**MSH2**	Cell Signalling Technologies	#2017	1:500 (ICC), 1:1000 (WB)
**MSH3**	ProteinTech	22393-1-AP	1:500 (ICC), 1:1000 (WB)
**MSH6**	BD	610918	1:500 (ICC), 1:1000 (WB)
**LIG1**	ProteinTech	18051-1-AP	1:500 (ICC), 1:1000 (WB)
**FAN1**	University of Dundee / CHDI	FS2	1:2000 (WB)
**EXO1**	ProteinTech	16253-1-AP	1:2000 (WB)
**LIG4**	ProteinTech	12695-1-AP	1:2000 (WB)
**ACTB**	Abcam	ab8226	1:5000 (WB)
**TUB**	Abcam	ab6046	1:5000 (WB)
**GAPDH**	Abcam	ab9485	1:5000 (WB)
**CTIP2**	Abcam	ab18465	1:500 (ICC), 1:1000 (WB)
**DARPP-32**	Cell Signalling Technologies	#2306	1:500 (ICC)
**MAP2**	Novus	NB300-213	1:10,000 (ICC)
**NFL**	Cell Signalling Technologies	#2837	1:2000 (WB)
**TUBB3**	Abcam	ab41489	1:2000 (ICC)
**NEUN**	Abcam	ab104224	1:500 (ICC), 1:1000 (WB)
**PCNA**	Cell Signalling Technologies	#2586	1:500 (ICC), 1:2000 (WB)
**OCT4**	Santa Cruz	sc-5279	1:1000 (ICC), 1:1000 (WB)
**NANOG**	abcam	ab21624	1:1000 (ICC)
**SSEA4**	Invitrogen	MA1-021	1:1000 (ICC)
**LIN28**	Cell Signalling Technologies	#3978	1:1000 (ICC)

Secondary	Supplier	Cat#	Dilution
**IRDye 800CW Goat anti-Mouse (H+L)**	LiCor	926-32210	1:15000
**IRDye 680RD Goat anti-Rabbit (H+L)**	LiCor	926-68071	1:15000
**AlexaFluor 488 Gt α Ms**	Invitrogen	A-11001	1:2000
**AlexaFluor 594 Gt α Rbt**	Invitrogen	A-11008	1:2000
**AlexaFluor 488 Gt α Rbt**	Invitrogen	A-11012	1:2000
**AlexaFluor 568 Gt α Rbt**	Invitrogen	A-11011	1:2000
**AlexaFluor 488 Gt α Rt**	Invitrogen	A-11006	1:2000
**AlexaFluor 647 Dk α Ms**	Invitrogen	A-31571	1:2000
**Hoechst 33342**	Invitrogen	H3570	1:2000
